# Elastic Composites Containing Carbonous Fillers Functionalized by Ionic Liquid: Viscoelastic Properties

**DOI:** 10.3390/polym17243271

**Published:** 2025-12-09

**Authors:** Magdalena Gaca, Magdalena Lipińska

**Affiliations:** Department of Chemistry, Institute of Polymer and Dye Technology, Lodz University of Technology, 16 Stefanowskiego Street, 90-537 Lodz, Poland

**Keywords:** carbon fillers, polymer composites, viscoelastic properties

## Abstract

In this work, the properties of polymer composites filled with carbon fillers were investigated. The subject of the research was polymeric materials prepared from styrene-butadiene rubber (KER 1500) commonly used in rubber processing, using a conventional sulfur-containing curing system. Two different carbon fillers were applied, namely furnace carbon black (N550) and graphene nanoplatelets (XG G300). These fillers were modified in bulk (during rubber compound preparation) with 4-methyl-1-butylpyridinium bromide (BmPyBr). Modifier would interact with filler’s surface through, e.g., π–π interactions between its pyridine ring and surface of the fillers. The paper highlights the different tendency of the polymer to interact with filler particles of different shapes and sizes, as well as the interactions between filler particles in the presence of an ionic liquid. The rheometric properties of rubber compounds as well as cross-linking density and mechanical properties of SBR composites were studied. Additionally, rheological and viscoelastic properties at the service temperature and the damping properties as a function of deformation of the obtained materials were examined.

## 1. Introduction

The processing of polymeric materials is significantly dependent on fillers dispersed in the polymer matrix. The usefulness of elastomer compositions without any fillers is small because these additives are added into the polymer matrix in order to improve composites’ properties or reduce costs of their production or final product. The most commonly used fillers are finely crushed mineral substances such as silica, chalk and montmorillonite [1,2,3,4,5,6,7]. Apart from them, fillers obtained from waste have been very popular in recent years. These include bamboo, wheat straw, rice husks and flax fibers [8,9,10,11,12,13,14]. The use of renewable raw materials and agricultural waste reduces dependence on fossil raw materials, which are used to produce, for example, carbon black, which is commonly used in polymer processing [15,16,17,18]. Thus, a sustainable development strategy contributes to the production of more environmentally friendly materials.

The basic parameters influencing the performance of fillers in polymers are particle size, their specific surface area and the ability to create their own “structure” in the polymer [19,20,21,22]. The aspect ratio of a filler, defined as the ratio of its length to its diameter, determines the properties of polymer composites. It is especially pronounced in the case of fibers or long particles characterized by high aspect ratio [23,24,25].

The surface activity of fillers, i.e., their chemical nature and energy state (surface energy, distribution of energy centers, structural defects and impurities) also plays an important role [26,27]. The interactions at the polymer–filler interface or between filler molecules are closely related to the surface activity of the filler [28].

From the point of view of the properties of filled polymer composites, an important issue is the homogeneous dispersion of the filler in the polymer matrix. The ability of the filler to create its own “structure” in the polymer and the lack of agglomeration of its particles in the polymer are crucial for obtaining composites with better mechanical and rheological properties [29].

To date, much research has been devoted to improving the dispersion of fillers in polymers using various methods. Ultrasonic homogenization has been used extensively; however, it leads to the destruction of weak bonds between filler molecules. Functionalization of the filler surface by depositing low-molecular-weight compounds or moderating filler–polymer interactions using various chemical compounds has been described in the literature [30,31,32]. It turned out that the solutions proposed to improve the degree of dispersion of fillers in the polymer led to an increase in the efficiency of stress transfer from the polymer matrix to the dispersed phase, which translated into an improvement in the strength properties of the obtained polymer composites.

Ionic liquids (ILs) are composed of specific organic anions and inorganic or organic cations [33]. These salts have low viscosity and saturation vapor pressure and are excellent solvents. The development of ILs’ research deals not only with their synthesis but also the possibilities of their application. It is known that ILs can be easily recycled; therefore, their use in industry is associated with a reduction in production costs. The occurrence of a wide range of these compounds plays a significant role in the spread of ILs. Various organic cations, including those with different substituents, can be successfully combined with any anions. In this way, ILs are designed to meet many specific requirements. Due to unique properties, ILs are used, e.g., in catalysts [34], chemical synthesis [35], sensors [36] and polymer materials [22,37,38,39]. When used in polymer composites, ILs play the role of solvents, ingredients of curing system, plasticizers, grafting agents or compounds modifying polymer fillers.

Generally, filled rubbers are characterized by complex viscoelastic characteristics. This means that their behavior under the influence of applied stress and strain changes with time, temperature and strain amplitude. Due to the fillers present in the polymer matrix, the mobility of their chains changes and polymer–filler and filler–filler interactions occur, which influence the storage modulus and loss tangent of polymeric materials. It has been proven that the intensity of the Payne effect observed in polymer composites depends on the type, structure and properties of the filler used in the composite [40,41]. Furthermore, the dynamic viscoelastic behavior of filled elastomers is dependent on the tendency to create filler–filler interactions in polymer materials [42].

For this research, two carbon fillers with different aspect ratios were selected and blended with SBR. The carbon black (CB) and graphene nanoplatelets (GnPs) were investigated. GnPs were chosen because they are very challenging (due to their tendency to agglomerate particles within a polymer) but also a promising polymer filler (their potential applications in smart polymer materials have been confirmed in many studies). Our previous research on the use of GnPs in elastomeric sensors [43,44] prompted us to undertake work on the viscoelastic properties of rubber composites containing this filler and ionic liquid. In turn, CB is the most commonly used filler in rubber processing, with a different size and shape of particles than GnPs. Although in practice the amount of CB in rubber products can reach several dozen of phr, in these studies, it did not exceed 5 phr and was treated as a reference rubber ingredient. The low amounts of proposed fillers were in accordance with the industrial trends in elastomers’ processing [45]. By using a low-molecular-weight compound (ionic liquid), the interactions between polymer macromolecules and the filler, as well as between filler particles, were influenced. In the obtained composite, pyridine derivative would interact with the surface of CB or GnPs through, e.g., π–π interactions between the pyridine ring and the surface of the fillers.

The strategy of surface modification of fillers was implemented by introducing these fillers and the modifier directly into the rubber compound during its preparation (modification in bulk). This method of filler modification did not involve additional technological steps, e.g., fillers immersing in solvents, solvents evaporation or filler drying, which would be expensive, material- and time-consuming. Moreover, this approach is an ecological solution implemented without the use of toxic solvents.

In this way, it was possible to investigate the effect of ionic liquid-modified fillers with different particle shapes on the viscoelastic properties of cross-linked elastomers and uncured rubber compounds. Additionally, mechanical properties of the obtained SBR composites were analyzed. The collected research results will be a valuable tool in the design of functional polymer composites.

## 2. Materials and Methods

### 2.1. Materials

**Figure 1:**
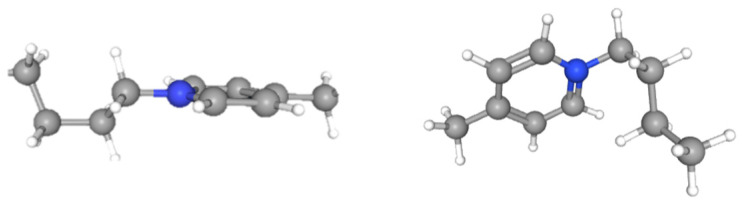
Models of chemical 3D structure of BmPyBr [43,44].

SBR (KER 1500) was supplied by Synthos S.A., Oswiecim, Poland. Both accelerators, namely 1,3-diphenylguanidine (DPG) and 2,2′-dibenzothiazyl disulfide (MBTS) were provided by Akrochem Co., Akron, OH, USA. Sulfur was supplied by Siarkopol, Tarnobrzeg, Poland. Graphene nanoplatelets powder (GnPs) in a high-purity form was purchased from XG Sciences Inc., Lansing, MI, USA. Carbon black (FEF N-550) was provided by Konimpex Sp z o.o., Koło, Poland. 4-methyl-1-butylpyridinium bromide (BmPyBr) was supplied by IoLiTec Ionic Liquids Technologies GmbH, Heilbronn, Germany [46,47]. Models of 3D structure of BmPyBr are presented in Figure 1.

The cross-linking substance (sulfur) and curing accelerators (DPG and MBTS) that were chosen in these studies are the typical cross-linking system used in the rubber industry for SBR vulcanization.

### 2.2. Preparation of Rubber Mixes and Vulcanizates

The rubber compounds were processed when the initial temperature of rolls of two roll-mill was set to 40 °C. This allowed the rubber to be masticated for 4 min before the next rubber compounds’ ingredients were introduced. Next, compounding additives were incorporated sequentially as follows: ionic liquid, filler (CB or GnPs), MBTS and DPG and finally sulfur. After 10 min of mixing, SBR compounds were finished (in a form of rubber sheets).

The experimental formulas of the filled rubber compounds and the reference sample are shown in Table 1. The amount of BMBP was equal to 1 mmol of ionic liquid per 1 g of filler in each mix.

**Scheme 1:**
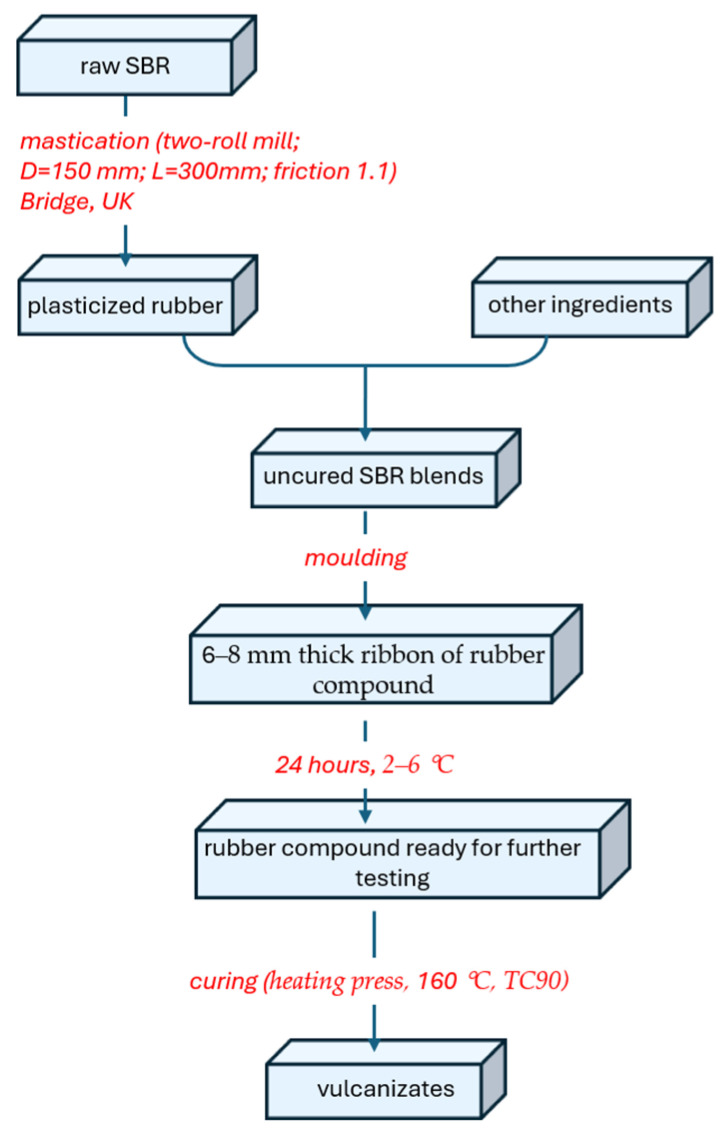
Strategy of rubber compound and vulcanizates’ preparation.

All vulcanizates were prepared according to the protocol drawn in Scheme 1.

### 2.3. Methods of Characterization

Attenuated total reflection Fourier transform infrared (ATR-FTIR) was applied to prove the interactions between pyridinium ring of BmPyBr and the surface of both carbon fillers. All spectra were recorded using a Thermo Scientific Nicolet 6700 FT-IR spectrometer (Waltham, MA, USA). Data were collected in absorbance mode with 64 scans and 4 cm^−1^ of resolution.

To estimate the surface free energy (*γ*) of the fillers before and after BmPyBr treatment, the Owens–Wendt–Rabel–Kaelble (OWRK) method was used [48,49]. The polar (*γ_p_*) and dispersive (*γ_d_*) components of *γ* were evaluated. For this purpose, fillers were wetted with 1,4-dioxane or dimethyl sulfoxide. A tensiometer K100 MKII (Kruss GmbH, Hamburg, Germany) was used during the dynamic tests of capillary wicking. All measurements were carried out at room temperatures and with n-hexane as a reference. The influence of BmPyBr on GnPs and CB surface properties was studied for both powdered fillers, pristine or carefully mixed with BmPyBr (each time, the ratio of filler to BmPyBr was 1 g to 1 mmol).

Rotational rheological measurements were carried out for the dispersion of GnPs and CB in the presence of BmPyBr in dibutyl phthalate (DBP) at concentrations of 5% and 50%. An oscillation rotational rheometer ARES G2 (TA Instruments, New Castel, DE, USA) and plate–plate geometry with a diameter of 25 mm were used during all tests. Before the measurement filler dispersion in DBP was ultrasonically treated during 10 min, hysteresis loop of shear viscosity was measured. For this purpose, each measurement was carried out at ambient temperature using a two-step procedure; in the first step, the applied shear rate increased in the range of 0.01÷0 s^−1^ for 60 s; in the second step, the applied shear rate decreased in the range of 20÷0.01 s^−1^. The area under the viscosity plots was determined and the area of the hysteresis loop was calculated.

The curing performance of the SBR compounds were studied using rotational rotorless rheometer D-RPA 3000 (MonTech, Columbia City, IN, USA), according to the standard [50]. The torque moment as a function of the vulcanization time at a constant frequency (1.7 ± 0.1 Hz), temperature (160 °C) and amplitude of oscillation (3°) was measured for all samples. Based on these measurements, the following parameters were determined: the minimal and maximal modulus (respectively, *M_L_* and *M_H_*), the induction time (*t*_Δ2_), the optimal curing time (*τ90*). The curing rate index (*CRI*) was calculated using the following equations (Equation (1)):

(1) $$CRI=100/(\tau 90-t_{\Delta 2})$$

To estimate the influence of BmPyBr on SBR compounds’ curing, some kinetic parameters were calculated. During the tests, the frequency 1.667 Hz and angle 0.5° were used. The state of cure is a function of reaction time and can be described by Equation (2):

(2) $$\frac{d\alpha }{dt}=K\;f\left(\alpha \right)$$

where *α* is the dimensionless fraction of reaction (conversion 0 < *α*, 1), *t* is reaction time, *K* is rate constant at a particular temperature. Using rheometric experimental data, the degree of vulcanization *α* at time t can be calculated according to the following Equation (3) [51]:

(3) $$\alpha \;\left(t\right)=\frac{M_{t}\;-M_{0}}{M_{\infty }-M_{0}}\;$$

where *M*_0_, *M*_∞_. *M_t_* are the elastic torque at the beginning of curing at *t* = 0, at the end of curing reactions, and at current time t., respectively.

Another empirical kinetic model was proposed to express *α* as a function of time (Equation (4)) [52]:

(4) $$\frac{d\alpha }{dt}=\frac{n}{K}t^{-1-n{\;\alpha }^{\;2}}\;$$

By taking *α* with respect to time, the equation can be expressed as Equation (5):

(5) $$\alpha =\frac{K\;t^{n}}{1+K\;t^{n}}$$

and can be further rearranged to Equation (6):

(6) $$\ln\left(\;\frac{\alpha }{1-\alpha }\;\right)=n\;lnK+\ln K$$

The rheometric data was fitted to the above equation. *K* and *n* (meaning the order of reaction) were calculated. According to the Arrhenius equations (Equation (7)), the apparent activation energy of rubber compound curing (*E_a_*) was calculated:

(7) $$K=k_{0}\exp(-\;\frac{E_{a}}{RT})$$

where *k*_0_ is the pre-exponential factor, *R* is universal gas constant (equal to 8.31447 J mole^−1^ K^−1^) and *T* is absolute temperature.

This apparatus was also used to study viscosity and viscoelastic properties of uncured rubber mixtures according to ASTM D6204 standard [53]. Before the tests, samples were thermally stabilized at 80 °C for 1 min without any applied strain. Various types of tests were performed for plasticized rubber compounds and different data was obtained: (a) the maximum values and the decrease in the dynamic (*η*′) and complex viscosity (η*) determined as a function of 2 min time of plasticization, during tests, the applied equivalent of shear rate was 2.5 s^−1^, (b) the dynamic (*η*′) and complex viscosity (η*) determined as a function of shear rate in range 0.5–315 s^−1^, (c) the average values of storage shear modulus (*G*′*_LVR_*), loss shear modulus (G′„_LVR_) and tan*δ*_LVR_ determined for linear viscoelastic region (during tests, the frequency 0.5 Hz and oscillation strain 0.2% were applied).

Dynamic RPA rubber process analyzer is a useful tool to study the viscoelastic behavior of rubber compounds before curing. Using the complex torque response (S*), the phase angle *δ*, the viscous torque (*S*′„) (out-of-phase with applied strain) and the elastic torque S′ (in phase with the applied strain), the viscoelastic parameters, storage modulus (*G*′), loss modulus (*G*′„) and loss factor (tan*δ*) can be easily derived according to Equations (1)–(4) [54].

The storage shear modulus (*G*′)′ and loss shear modulus (*G*′„) can be calculated according to Equations (8) and (9):

(8) $$G^{\prime }=\frac{k\cdot S^{\prime }}{applied\;strain}$$

(9) $$G^{\prime \prime }=\frac{k\cdot S^{\prime \prime }}{applied\;strain}$$

where *S*′—elastic torque, *S*′„—viscous torque, *k*—constant, taking into account the geometry of the die cavity of the testing equipment.

The complex viscosity (η*) and the dynamic viscosity (*η*′) were calculated according to Equations (10) and (11):

(10) $$\eta^{*}=\frac{G^{*}}{\omega }$$

(11) $$\eta^{\prime }=\frac{G^{\prime \prime }}{\omega }\;$$

where *ω*—angular frequency in rads^−1^, G*—complex shear modulus were defined as follows (Equation (12)):

(12) G*=[(G′)2+(G″)2]12

For cured rubber samples (at heating hydraulic press at 160 °C under 15 MPa pressure), the following tests were performed.

The cross-linking density of cured samples (*ν*) was determined using the Flory–Rehner equation (Equation (13)) [55].

(13) $$\nu =-\frac{\ln\left(1-Vr\right)+Vr+\chi {Vr}^{2}}{Vs\left({Vr}^{1/3}-\frac{1}{2}Vr\right)}$$

For this purpose, small pieces of vulcanizates with known weights were swollen in toluene for 48 h at room temperature. Then, the solvent was removed and the samples were weighed, after which they were dried at 50 °C for two days. Next, the dried samples were reweighed. In Equation (7), *Vr* is the volume of the elastomer fraction in swollen gel, Vs is the molar volume of solvent and *χ* is the Huggins parameter of SBR–toluene interaction given by Equation (14):

χ = 0.0501 + 1.9694*Vr* (14)

To assess the presence of noncovalent cross-links in composite materials, a similar procedure was used. This time, the samples were swelled in toluene under ammonia-saturated vapor in desiccator at the room temperature for a period of 48 h (*ν_A_*). On this basis, the concentration of these specific links (Δ*ν*) was estimated from the difference between *ν* and *ν*_A_. It was discussed elsewhere that NH_3_ contributes to the disintegration of the links formed at the filler–rubber interfaces [56].

The tensile testing was performed according to the standard ISO 37-2017 [57], with a universal testing machine (Zwick, model 1435, ZwickRoell GmbH, Ulm, Germany), operated at a constant crosshead speed of 500 mm min^−1^ at room temperature. At least 5 samples of a dumbbell-shaped thin strip cut with a razor blade were tested for each vulcanizate. All surfaces of the test specimens were without visible imperfections (e.g., flaws or scratches).

Additionally, the reinforcing index *RI* proposed by Kohjiya and Ikeda was calculated according to the equation below (Equation (15)) [58]:

(15) $$RI=\frac{N}{N_{0}}\cdot \frac{m_{F}}{100}$$

where *N*, *N*_0_ are values of selected mechanical parameters, e.g., tensile strength for vulcanizates filled and referenced (unfilled), respectively, *m_F_*—mass of filler added.

The activity of the fillers used was indicated by the steepness of the strain–stress curves and expressed by dependence SE300/SE100 [59].

The relaxation behavior of filled composites was studied at room temperature using a universal testing machine (Zwick, model 1435, ZwickRoell GmbH, Ulm, Germany) at a constant strain level of 100%. Then, the movement of the grip was halted and the decrease in stress with time was recorded for a period of 30 min.

Relaxation behavior of samples containing 3 or 5 phr of the filler was studied under shear deformation at a constant strain level of 5% during 30 min using Ares G2 equipment. The values of modulus *G* determined as a function of time were fitted to the generalized Maxwell model according to Equation (16) [60,61]:

(16) Gt=Ge+∑iGie−tτi

where *G*(*t*) is stress relaxation modulus at time *t*, *G_e_* is the equilibrium modulus representing stress that remains after a very long time, *G_i_* are the Prony series coefficients (the relaxation moduli) and *t_I_* are the relaxation times for each *i*-term in Prony series. Each term describes a different relaxation process occurring in the studied sample, *i* is the number of terms in the series, which corresponds to the number of Maxwell elements in the generalized Maxwell models. The series of *i* = 4 and *i* = 5 Prony elements were used during fitting. TA Instrument Trios software (TRIOS^®^ Software v3 1.5.3696) was used for Prony series analysis.

The viscoelastic properties of cured rubber, namely, storage shear modulus (*G*′), loss shear modulus (*G*′„) and loss factor (tan*δ*) were determined using rotational oscillation rheometer Ares G2 (TA Instruments, New Castle, DE, USA) equipped with parallel plate–plate geometry with diameter 25 mm. The amplitude sweep tests at room temperature 25 °C were performed at angular frequency 10 rads^−1^. The average values of viscoelastic parameters were calculated for linear viscoelastic region LVR from the oscillation strain range 0.01–0.1%. To analyze the Payne’s effect [62], the amplitude sweep tests (temperature 25 °C, angular frequency 10 rads^−1^) were performed in two cycles, and the oscillation strain range in range 0.01–150% was applied during both cycles. To estimate the influence of ionic liquids and fillers on the glass transition *T_g_* of SBR rubber, the oscillation sweep test in temperature range from 20 °C to −80 °C with cooling rate 5 °C min^−1^ at constant frequency of 1 Hz and constant oscillation strain of 0.1% were conducted.

Scanning electron microscopy (SEM, Zeiss Ultra Plus, Carl Zeiss MD GmbH, Oberkochen, Germany) was adopted to investigate the dispersion of CB or GnPs. Strips of the vulcanizate were immersed in liquid nitrogen to freeze it for 1 min. Afterwards, the sample was fractured. The fractured surface was coated in a thin layer of conductive powder. Electron images of such fractured surface were taken for all filled vulcanizates with an operating voltage of 3 kV at various levels of magnification. Additionally, the quantitative image analysis was performed using ImageJ v1.54p software.

The amount of the bound rubber (*R_B_*) in uncured filled rubber compounds was determined by the extraction of it from these rubber compounds with the use of toluene during 144 h at room temperature. The procedure was as follows: a piece of rubber compound was wrapped in a filter paper and poured with 100 mL of solvent. After 6 days, toluene was replaced with acetone for 24 h. Then samples were dried and weighted. Next, the percent-bound rubber of the polymer (*R_B_*) was calculated according to Equation (17):

(17) $$R_{B}=\frac{a-b[\frac{c}{c+d}]}{b\;[\frac{d}{c+d}]}\times 100$$

where *a* is the weight of filler and gel, *b*, is the weight of rubber compound, and *c* and *d* are, respectively, the weights of filler and rubber in studied rubber compound.

## 3. Results and Discussion

### 3.1. Properties of CB and GnPs

In this paper, fillers’ modification in bulk was proposed. This made it difficult to study properties of only the fillers in the polymer. To prove modification of the fillers with the use of BmPyBr, the surface energy of these powdered fillers was estimated (Table 2).

GnPs were characterized by lower *γ_s_* when compared with CB (20.7 and 30.2 mN m^−1^ for GnPs and 29.1 and 35.4 mN m^−1^, in the case of CB). In the absence of BmPyBr, CB was found to have higher *γ_s_* than GnPs. A similar tendency was shown for both fillers modified with BmPyBr. For both fillers modified with BmPyBr, an increase in *γ_s_* was confirmed. Then, *γ_s_* increased from 30.2 mN m^−1^ for G_IL to 35.4 mN m^−1^ for CB_IL. Before modification, GnPs and CB had reduced polar component of *γ_s_*. The occurrence of interactions between the fillers and BmPyBr may be confirmed by the increase in polarity of these fillers in the presence of BmPyBr (in the case of GnPs, from 0.1 to 5.6 mN m^−1^, and for CB, *γ_p_* yielded 1.2 mN m^−1^ and 10.7 mN m^−1^).

Fillers’ modification with BmPyBr and interactions between the planar part of ionic liquid and GnPs were proved by ATR-IR spectroscopy (Figure S1). Neat GnPs or CB did not show any significant absorption band. When fillers were mixed with BmPyBr, a small peak corresponding to NH-stretching vibrations in amine salts were noticed above 2950 cm^−1^. The presence of pyridine ring was indicated by a small peak (approx. 1450 cm^−1^). CN-bond stretching was shown as the peak at approximately 1180 cm^−1^. Both the cation and the anion of BmPyBr can interact with the filler surface [44]. However, previous studies revealed that intermolecular interactions are predominantly provided through cation–π interactions [44]. Hence, the results we obtained are consistent with the studies relating to carbon fillers modified with ionic liquids in a different way, namely by depositing them on the surface of fillers from a solution.

### 3.2. Viscoelastic Properties and Viscosity of Uncured Rubber Compounds

The dynamic (*η*′) and complex viscosity (η*) of rubber compounds were studied as a function of time and the results are shown in Figures S2–S5. These parameters are analogous of the apparent viscosity (*η_app_*) and viscosity corrected from the capillary rheometers [63].

The values of viscosity, as measured at the beginning of test at time 2 s, and the decrease in the viscosity after 2 min of applied strain, equivalent of the 0.1 s^−1^ shear rate, are compiled in Table 3. The increase in the initial viscosity of uncured but filled rubber compounds can result from three facts, these are (a) the hydrodynamic effect of the filler, (b) the interactions between filler surface and polymer chains and (c) the interactions among filler particles alone [64]. As it was expected, the incorporation of a higher amount of GnPs or CB led to an increase in maximum value of η* at the beginning of the test. BmPyBr, in most cases, caused further increase in both parameters, respectively, η* and *η*′. This effect was dependent on the dispersion of filler particles within polymer and the influence of the ionic liquid on it. The viscosity gradually decreased from its maximum value as the measurement time was lengthened. Hence, the shear rate and strain applied to the sample caused reorganization of the alignment of the solid particles in plasticization during measurement of rubber, thus leading to the lower and then constant values of viscosity. The highest Δη* (difference between the maximum and minimum value of viscosity) was observed for the sample containing 3 phr of GnPs and BmPyBr. This indicated the strongest structurization of the filler in rubber.

Additionally, the changes in η* as a function of shear rate were analyzed (Figures S6 and S7). Molten rubber compounds are shear-thinning, non-Newtonian fluids whose viscosity is described by a power law model [63]. The obtained data were fitted to the power law equation. The values of the consistency index *K* and the exponent of potential equation *n* were calculated according to (Equation (18)):

(18) $$\eta =K{\dot{\gamma }}^{n}$$

The values of parameters *K* and *n*, together with the values of η* at shear rate of 300 s^−1^, are compiled in Table 4. All rubber mixtures were characterized by decreasing η* as an applied shear rate was higher and the exponent of potential equation in the range between −0.763 and −0.730. Here, the stronger reduction in viscosity (when the shear rate increased) was observed for the rubber compound with 5 phr of GnPs and BmPyBr (*n* = −0.763) rather than for the sample with 5 phr of CB and BmPyBr (*n* = −0.730). At a shear rate of 300 s^−1^, characteristic of industrial processing methods such as high shear extrusion, the viscosity of all rubber mixtures was in the range from 589 to 687 Pas. The incorporation of fillers in the form of solid particles increased the consistency index *K*. For rubber compounds containing GnPs, such impact was stronger. In most cases, the addition of ionic liquid to the rubber compound resulted in a stronger shear thinning of the rubber compound, as evidenced by lower η* in the shear range above 100 s^−1^. This behavior is an advantage in rubber processing.

The viscoelastic properties of uncured rubbers at processing temperature of 80 °C were analyzed for the linear viscoelastic range. In those conditions, the applied level of deformation (0.2%) and frequency (0.5 Hz) did not influence the viscoelastic properties. The values of storage shear and loss shear moduli (respectively, G′ and G″) and loss factor (tan*δ*) of rubber compounds are shown in Table 5. Uncured rubber has elastic properties, primarily due to the presence of polymer chain entanglements. So, it is possible for various rubber compounds to have the same viscosity but different levels of elasticity. Higher values of G′ (at defined temperature) can cause stronger die swell and poorer dimensional stability during downstream extrusion process [63]. Dominant elastic behavior was observed for all rubber compounds plasticized at 80 °C, with G′ being higher than G′„ and loss tangent being less than 1. The filled rubber compounds were characterized by enhanced G′ compared to the unfilled reference rubber compound, which was more evident in the case of GnPs. Further, the introduction of BmPyBr to rubber compounds led to additional increase in G′. It is attributed to better dispersion of CB or GnPs in the presence of BmPyBr. Stronger dissipation of the deformation energy was related to the enlarged G′„ of samples filled with solid particles.

**Figure 2:**
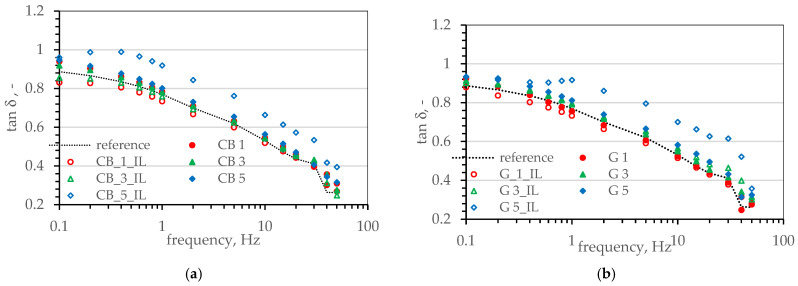
tan*δ* values of SBR rubber compounds measured at 80 °C under oscillation deformation of 7%: samples containing CB (**a**) or GnPs (**b**).

To deepen the research on rubber compounds, their viscoelastic properties were studied as a function of frequency at a higher value of oscillation strain equal to 7%. It is important to note that similar ratio of oscillation strain is used during further described tests of curing performance of SBR compounds. G′ and G′„ are shown in the Supporting Information (Figures S8 and S9). The values of tan*δ* were calculated according to the following equation (Equation (19)) and are depicted in Figure 2:

(19) $$\tan\delta =\frac{G^{\prime \prime }}{G^{\prime }}$$

The higher tan*δ* values of rubber compounds implies a higher ratio of viscous to elastic properties. The parameter tan*δ* was found to be at least twice as sensitive to real differences in rubber processing than the α slope from the Mooney stress relaxation test [63]. Therefore, tan*δ* together with *G*′ can be a useful parameter to compare the differences in processing of various uncured rubber mixtures at selected processing temperature. It is strongly visible (Figure 2) that the addition of BmPyBr facilitated the processing of rubber mixtures containing 5 phr of the filler. This led to significantly higher values of tan*δ* in the whole studied frequency range both in CB and GnPs. This is beneficial from the point of view of processing of these rubber compounds.

### 3.3. Properties of SBR Composites

**Figure 3:**
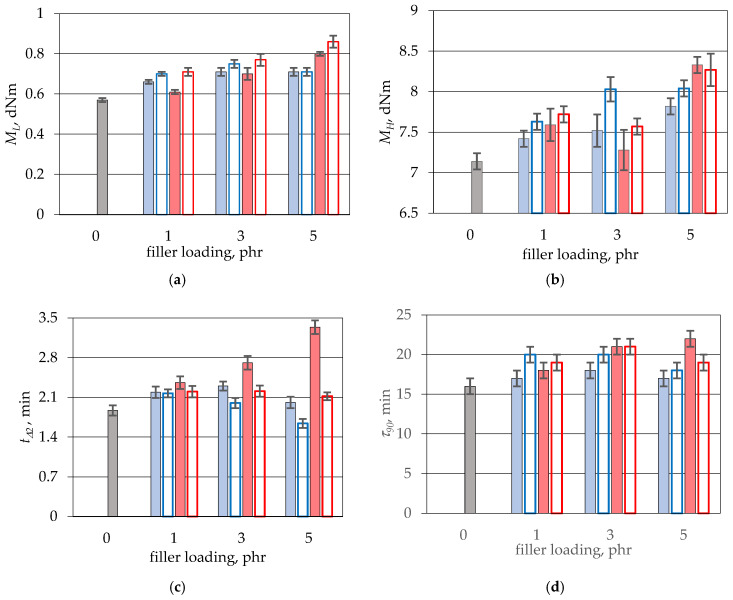
Curing characteristics of unfilled and filled rubber compounds: (**a**) minimum torque (*M_L_*), (**b**) maximum torque (*M_H_*), (**c**) induction time (*t*_Δ2_), (**d**) optimal curing time (*τ*_90_); gray bar—reference sample, blue bar—sample with CB, empty blue bar—sample with CB and BmPyBr, red bar—sample with GnPs, empty red bar—sample with GnPs and BmPyBr.

The cure behavior of the different compounds was investigated at 160 °C in a rotorless rheometer. The influence of both CB and GnPs loading and the presence of selected BmPyBr on the curing behavior was analyzed (Figure 3, Table S1). The parameters such as minimal value of elastic torque (*M_L_*), maximal value of elastic torque (*M_H_*), the increase in the elastic torque during curing (Δ*M*), the scorch time (*t*_Δ2_) (time required at specified temperature for rubber mixture to form initial stage of cross-links) and curing time (*τ*_90_) (time necessary to reach a 90% state of cure) were determined.

Incorporation of CB did not strongly affect *M_L_* (Figure 3a). Stronger influence on *M_L_* was observed after the addition of GnPs into SBR matrix (especially in the case of 5 phr filler loading). The higher aspect ratio of GnPs′ particles at similar filler loading had a stronger effect on the viscosity of rubber before curing due to the stronger hydrodynamic effect generated by solid particles. Mixtures containing both fillers and BmPyBr were characterized by similar Δ*M* (Table S1). This, in turn, indicated that the fillers and BmPyBr had no significant effect on the final state of cure. Further, the calculated values of “total” cross-link density of the vulcanized SBR samples were not meaningfully different, confirming that the addition of BmPyBr did not affect the amount of covalent cross-links formed in the 3D polymer network (Figure S10). The Flory–Rehner equation does not distinguish between chemical and physical cross-links in the polymer network. The cross-links of the elastomer network that do not have any covalent character were destroyed to some extent under ammonia treatment [56]. Therefore, the content of noncovalent cross-links in polymer network (Δ*ν*) was determined based on the equilibrium swelling of SBR composites in toluene upon NH_3_ vapors (Table S2). The obtained results are consistent with the bound rubber amount estimated for all filled SBR composites and discussed later.

BmPyBr slightly decreased *t*_Δ2_ of the samples when compared with rubber compounds without BmPyBr (Figure 3c). This confirms the accelerating effect of BmPyBr on the kinetic of sulfur curing of the rubber. Similar conclusions were also reported by other authors [65]. No significant effect of CB on *τ*_90_ of SBR was determined (Figure 3d). The use of CB in rubber compound extended their *τ*_90_ by up to 2 min compared to the unfilled rubber compound. In contrast, adding GnPs to rubber compounds resulted in a significant extension of their *τ*_90_. In the case of flattened GnPs particles, the curing components which are active in the vulcanization process and contain flat aromatic rings in their structure were preferentially adsorbed by the filler. Thus, their concentration in the polymer matrix was lowered, leading to lengthening of *τ*_90_ of rubber compounds. Such occurrence is disadvantageous from a practical, industrial point of view. The ingredients of the curing system may be adsorbed on the outer part of CB agglomerates due to steric hindrance of their aromatic rings [66]. Ionic liquids are known to be ingredients of rubber compounds that moderate kinetic of cross-linking (described in the literature [65]). Besides the catalytic effect of ionic liquids on rubber curing, these compounds can retard this process as well. Here, the incorporation of BmPyBr caused shortening of *t*_Δ2_ but made *τ*_90_ longer. This was particularly evident in the case of samples filled with GnPs. The probability of interaction of the pyridinium ring of BmPyBr with the aromatic and planar parts of (i) the used vulcanization accelerators and (ii) the filler was higher than in the case of CB.

**Figure 4:**
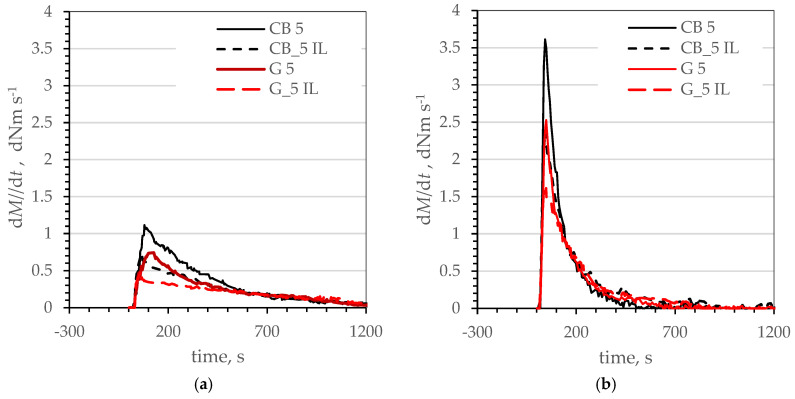
Cure rate (d*M*/d*t*) of rubber compounds containing 5 phr of CB or GnPs, measured at 160 °C (**a**) and 180 °C (**b**).

Additionally, we studied the curing of rubber compounds at various temperatures ranging from 160 to 180 °C (Figures S11–S14). Owing to this, deeper insight into the accelerating/retarding role of BmPyBr in curing process was gained. Analysis of curing rate in the presence of BmPyBr was also possible. The influence of both fillers, CB and GnPs, and ionic liquid on the first derivative of torque (*dM/dt*), so-called cure rate (*CR*), at 160 °C and 180 °C are presented in Figure 4. The values of *CR_max_* (at the maximum of the first derivative peak) were analyzed together with the values of cross-linking induction time (*t_i_*) determined for all studied temperatures (Table 6). Up to *t_i_*, the values of *dM/dt* (determined as a function of time) were equal to zero because no curing reactions occurred that could lead to the increase in *M* parameter. In the case of rubber compounds filled with CB and containing BmPyBr, a reduction in *t_i_* was observed at all tested temperatures. It proved a slightly accelerating effect of BmPyBr on the process of SBR compounds curing. When GnPs were used, the shortening of *t_i_* was less visible. Lower values of *CR_max_* of rubber compounds modified in bulk by BmPyBr certified that the presence of ionic liquid affected the process of their curing. Further, lower *CR* peaks were also observed for GnPs-SBR compounds at 160 °C and 180 °C, compared with CB-filled SBR.

In the case of the sample with BmPyBr and 5 phr of CB, higher *K* confirmed the accelerating effect of BmPyBr on curing of the SBR compound. Although the curing reaction was faster (for the particular temperatures), it was shown that *E_a_* was almost doubled. Similarly, the incorporation of BmPyBr into GnPs-filled SBR compounds led to the increase in *E_a_*. Due to interactions between BmPyBr and the surface of GnPs, the accelerating effect of ionic liquid on the curing process was reduced. This led to lower values of *K*.

**Figure 5:**
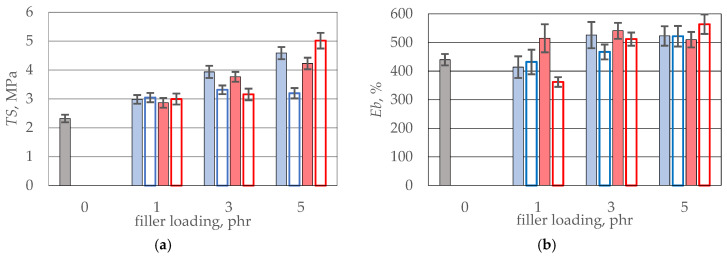
Tensile strength (*TS*) (**a**) and elongation at break (*Eb*) (**b**) of SBR composites: gray bar—reference sample, blue bar—sample with CB, empty blue bar—sample with CB and BmPyBr, red bar—sample with GnPs, empty red bar—sample with GnPs and BmPyBr.

Having established the influence of both active fillers on the vulcanization process and cross-link density of SBR composites, the static mechanical properties were examined as well (Figure 5 and Figure S15). Additionally, the reinforcing index (*RI_TS_*) was calculated according to the equation proposed previously [58] (see Table 7).

It can be clearly observed that the incorporation of GnPs together with BmPyBr at higher loading (equal to 5 phr) instead of CB had a stronger impact on *TS* of SBR vulcanizates, which manifested itself in a higher *RI_TS_* (Table 7). Next, a simple way to assess filler’s activity in the polymer matrix is by calculation of the steepness of mechanical curves characterizing tensile properties of filled elastomers at room temperature (*SE300/SE100*) [59]. *SE300/SE100* revealed that plate-like filler was even more active in SBR than CB (in the presence of BmPyBr) (Table 7).

**Figure 6:**
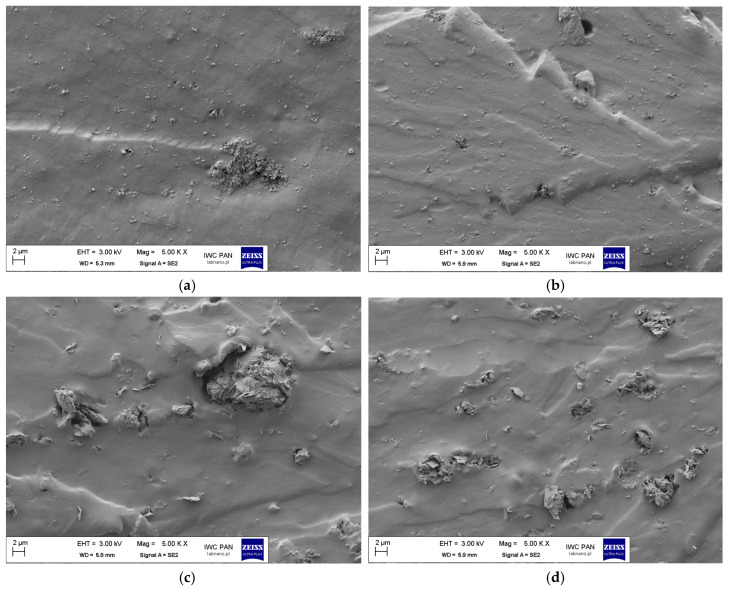
SEM micrographs of SBR composites filled with 5 phr of filler: CB (**a**), CB in the presence of BmPyBr (**b**), GnPs (**c**) and GnPs in the presence of BmPyBr (**d**).

Generally, reinforcing fillers are immiscible in the elastomer matrix, leading to the clustering of their particles and formation of larger agglomerates which can be detrimental for the properties of the final composite material [67]. Here, factors like good distribution of the filler in the polymer or the reduction in the size of its aggregates are important when discussing the reinforcing potential of the filler. The filler’s dispersion is one of several factors strongly influencing properties of cured rubber such as *TS*. The addition of BmPyBr can affect the mixing of GnPs and facilitates filler’s dispersion, as it was proven by the SEM analysis (Figure 6). It is not surprising that ionic liquids are known for improving filler’s dispersion [68]. In the case of composites with GnPs and BmPyBr, the size of filler’s aggregates was reduced. Based on SEM images, the average agglomerate size was quantitatively estimated and the results are presented in Table 8. It was clear that the use of BmPyBr in SBR composites had a positive effect on the dispersion of both filler particles in the polymer. This was evidenced by the reduction in the number of the largest filler agglomerates (from 13.4% to 5.8% for GnPs and from 9.1% to 3.2% for CB).

SEM images illustrated the presence of filler clusters that were raptured into a number of small agglomerates, leading to a reduction in the filler–filler interaction and an enhancement of the polymer–filler interaction. This phenomenon was in line with mechanical properties of the composites. Flat carbon sheets of GnPs were more likely to interact with BmPyBr (containing in its structure aromatic ring, thus being flat in some part) when compared with CB particles [69]. Due to more homogenous dispersion of filler particles in rubber composites, their load transfer efficiency can be enhanced [70].

No significant effect of the additives on SBR vulcanizates’ *Eb* was observed, although in the case of composites with GnPs it was slightly higher. All cured SBR composition had the value of *Eb* in the range of 400–550%, which is an optimal range for the industrial application of cured rubber.

To characterize SBR–filler interactions, influencing, e.g., rubber reinforcement, bound rubber content (*R_B_*) was calculated, and the results are presented in Table 9. The tested rubber mixtures differed not in the content of fillers but in the size of their particles. For all investigated samples, *R_B_* ranged from 1.06 to less than 6%. This indicated that a large part of the tested SBR compounds had been dissolved in toluene. When a small amount of the filler is added to rubber compound, the surface contact generated between polymer chains and filler surface is small. Thus, interactions between these two ingredients of the rubber compound are insufficient to create several dozen percent of *R_B_* [71]. The amount of filler proposed in these studies, not exceeding 5 phr, should be considered small from the point of view of polymer processing. This explains the relatively low values of *R_B_* in the case of the rubber compounds discussed here containing CB or GnPs. As CB’s loading increased (from 1 to 5 phr), the amount of bonds between the filler’s surface and SBR increased as well due to higher and higher concentration of active sites on the filler’s surface. This was manifested by increasing *R_B_*. An increase in GnPs’ concentration in rubber compounds promoted agglomeration of its particles in the polymer. Thus, *R_B_* of samples containing GnPs decreased with filler amount. It was found earlier that functional groups of the polymer had a positive effect on the formation of bonded rubber [71]. Owing to π-π interactions between phenyl groups of SBR and filler’s particles with delocalized electron clouds, *R_B_* of rubber compounds with GnPs was higher than for samples filled with CB. It was stated elsewhere that fillers with small particles form more bonded rubber [72]. This is consistent with the results obtained. GnPs with much smaller particles than CB have increased the formation of GnPs-SBR gel among studied rubber compounds.

When BmPyBr was added to the rubber compounds, the tendency to absorb the polymer onto the filler’s surface was different. Probably, ionic liquid blocked active sides on CB’s surface, preventing the absorption of polymer chains on its surface. It was evidenced by *R_B_* being lower than for CB-filled rubber compounds without BmPyBr. In turn, the pyridinium ring present in BmPyBr additionally supported the interactions of GnPs with SBR and *R_B_* enhancement.

**Figure 7:**
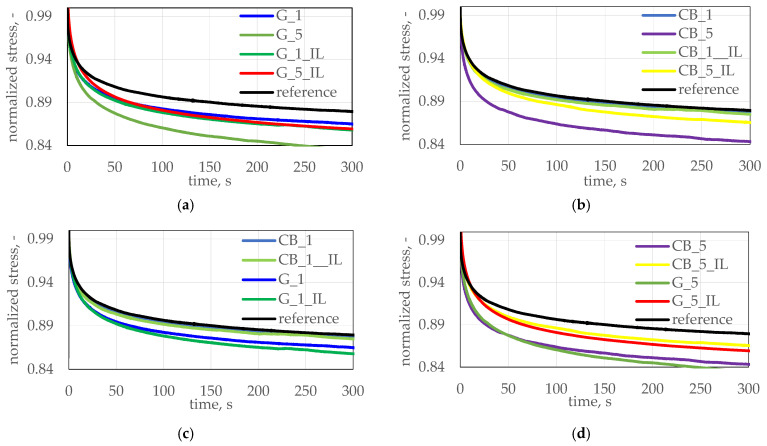
Time dependence of normalized stress (*σ_t_*/*σ_max_*) for polymer composites: (**a**) with different GnPs loading (1 and 5 phr of filler); (**b**) with different CB loading (1 and 5 phr of filler); (**c**) with 1 phr of filler; (**d**) with 5 phr of filler; the reference sample is added for comparison purposes.

The effect of both fillers having different aspect ratios on physical stress relaxation behavior of rubber composites at the constant strain (equal to 100% elongation) was investigated successfully. For each specimen, normalized stress (*σ_t_/σ_max_*) was plotted against time of samples relaxation. Data obtained on all samples is illustrated in Figure 7. The curves are all similar in shape; in the beginning, relaxation decays rapidly and then slowly. The presence of CB or GnPs in SBR composite may result in an increase in its relaxation rate [73]. Thus, it is not surprising that the sample of unfilled rubber tested in this work was characterized by the weakest physical stress relaxation (evidenced by the highest value of residual stress after the specified time had elapsed). Since physical relaxation of polymers depends on, among others, filler–filler and filler–polymer interactions, in the initial phase of this process, the interactions between the filler particles are weakened and decomposed. Then, the polymer–filler and polymer–polymer interactions are destroyed under the influence of the stress applied to the sample [74]. The study on the impact of filler’s loading on this phenomenon showed noticeable changes in the relaxation rate of the examined composites. At higher concentrations of GnPs or CB in the tested composites (equal to 5 phr), a larger drop in stress was observed due to the destruction of a significantly larger number of polymer–filler interactions. Similarly, the addition of BmPyBr had a beneficial effect on the physical relaxation of the tested composites.

The additional interactions between the particles of these fillers and BmPyBr promoted the reorientation of polymer chains, equalizing the lengths between the cross-links of their polymer networks, and thus increased the relaxation rate in the force field (Figure 7).

The physical relaxation of polymer composites attributed to disentangling of polymer chains from the polymer network, deformation and rupture of micro domains and cross-links was observed when composites were subjected not only to stretching but also to shear deformation. The rearrangement and motion of polymeric chains can be affected by the surrounding structures and various types of interface in polymer/filler systems (the degree of cross-linking and morphology of network). Moreover, nanoparticles added to the composites may strongly affect the transition of the system from one stage of thermodynamic equilibrium to another. Again, stress relaxation occurs usually according to multi-stage mechanisms in multiphase systems [73].

**Figure 8:**
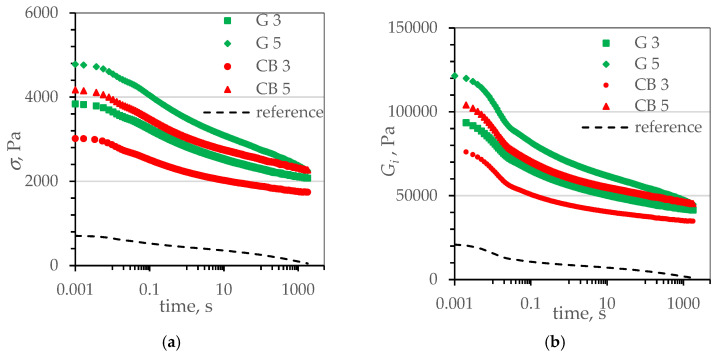
Stress relaxation (*σ*) (**a**) and shear relaxation modulus (*G_i_*) (**b**) of SBR composites containing GnPs or CB.

**Figure 9:**
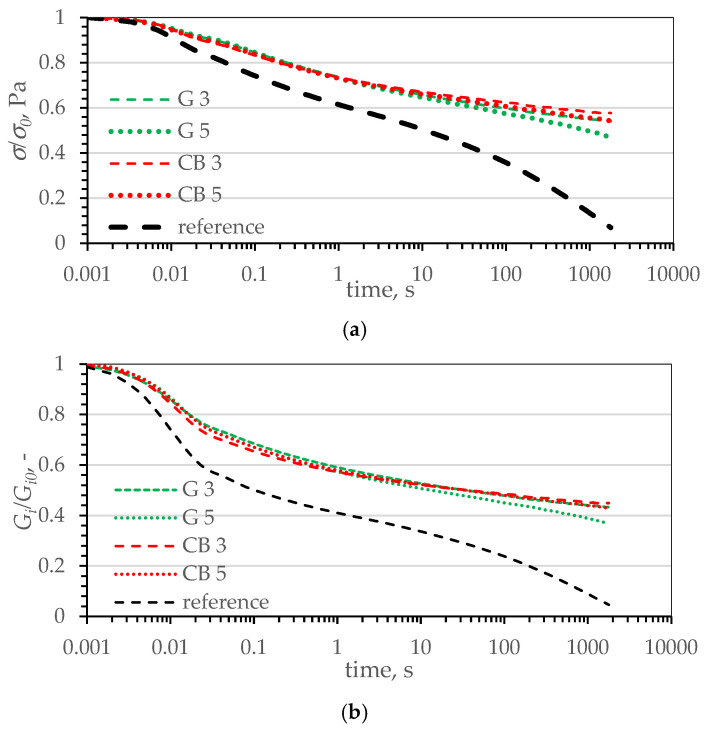
Normalized relaxation stress (*σ/σ*_0_) (**a**) and normalized relaxation modulus (*G_i_*/*G_i_*_0_) (**b**) for SBR composites containing CB and GnPs.

To illuminate diverse behavior of SBR composites under deformation, their relaxation was studied when 5% shear was maintained. The values of stress (*σ*) and relaxation modulus (*G_i_*) were recorded and determined during tests lasting 1800 s. First, samples filled with CB or GnPs were examined (Figure 8). During composite’s deformation, a part of the energy stored within is dissipated by the rearrangement of polymer network and some of the deformed chains cannot be retracted. Due to the reorientation of small segments of SBR chains at filler–polymer interface, the significant reduction in *σ* and rapid decrease in *G_i_* was noticed at the beginning of the test (time up to ~0.1 s). The longer time of relaxation was associated with the failure of elastomer–filler bonding and long-range rearrangement of the polymer chains. The flow of the matrix under shear at longer time caused the decrease in *σ* at the last decade of relaxation. A higher amount of fillers present in the composites led to increase in *G_i_*. A higher initial *σ* value was observed for samples containing GnPs, which can be attributed to stronger interactions at the filler–elastomer interface. In order to facilitate the comparison of relaxation between the tested composites, *σ* and *G_i_* were normalized with respect to the initial stress and relaxation modulus (*σ*_0_ and *G_i_*_0_, respectively) (see Figure 9). The residual stress (*σ*_1800_), characterizing all samples yielded at the end of the test, showed that the equilibrium was not reached during 1800 s. In the case of elastomer composites with CB, the lowest *σ*_1800_ was achieved for 3 phr of filler (equal to 1743 Pa), but the highest *σ*_1800_ was noticed for the sample containing 5 phr of CB (2269 Pa). A similar tendency was observed when composites were prepared with the use of GnPs instead of CB (for 3 phr of filler *σ*_1800_ = 2077 Pa and for 5 phr of GnPs *σ*_1800_ = 2245 Pa). Referring to the unfilled composite, no stress equilibrium state was observed and *σ*_1800_ corresponded to only 49 Pa.

**Figure 10:**
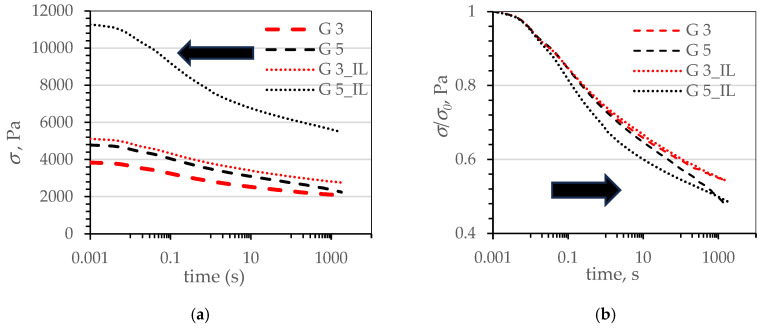
Stress relaxation (*σ*) (**a**) and normalized shear relaxation (*σ/σ*_0_) (**b**) of vulcanizates containing GnPs.

**Figure 11:**
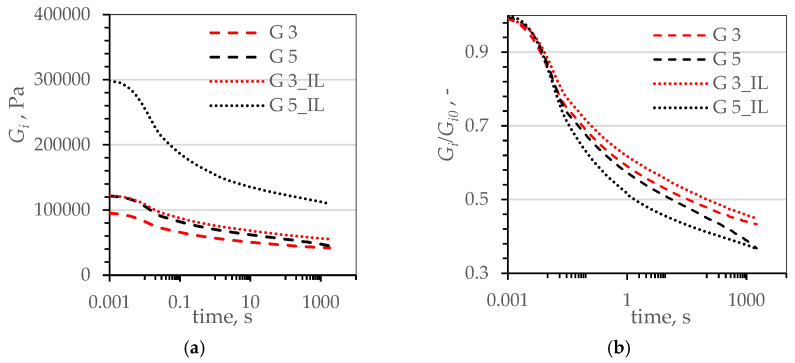
Relaxation modulus (*G_i_*) (**a**) and normalized relaxation modulus (*G_i_/G_i_*_0_) (**b**) of vulcanizates containing GnPs.

The relaxation behavior of samples containing BmPyBr was measured under shear (Figure 10). The addition of BmPyBr into GnPs-filled composites led to the significant increase in initial *σ* for both GnPs loadings. Probably, BmPyBr containing a flat part of molecule (relating to aromatic ring of pyridine derivative) is situated differently next to the carbon planes of GnPs and differently next to the spherical CB particles. When considering *σ*_1800_, its value is lower for samples containing 5 phr of GnPs with or without BmPyBr when compared with samples with 3 phr of filler. The incorporation of BmPyBr into SBR composite with 5 phr of GnPs facilitated the composite’s relaxation process at the very beginning of the test (time decade up to 1 s). Furthermore, in the case of that sample, at a longer time of relaxation, *G_i_* declined more slowly. This effect can be related to the interactions between aromatic rings of BmPyBr and carbon layers of GnPs (Figure 11).

**Figure 12:**
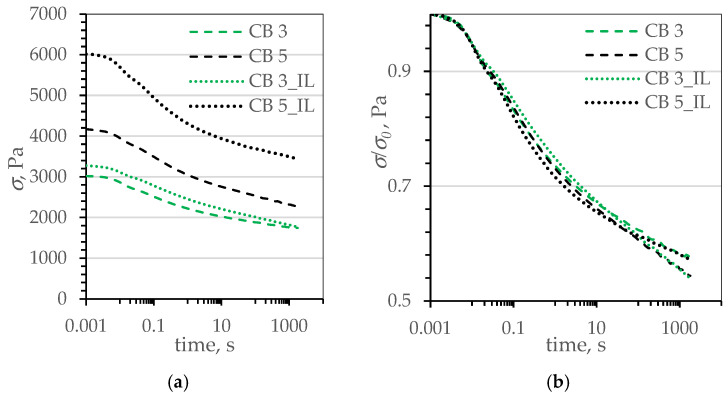
Stress relaxation (*σ*) (**a**) and normalized stress relaxation (*σ/σ*_0_) (**b**) of vulcanizates containing CB.

**Figure 13:**
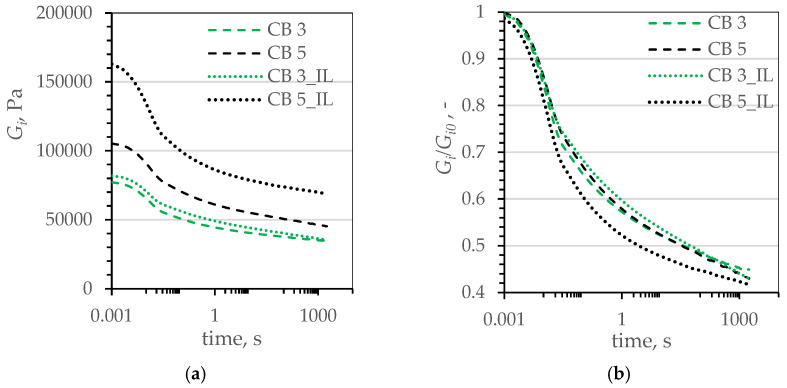
Relaxation modulus (*G_i_*) (**a**) and normalized shear relaxation modulus (*G_i_/G_i_*_0_) (**b**) of vulcanizates containing GnPs.

Relaxation behavior was also studied for the samples containing CB (Figure 12 and Figure 13). The addition of BmPyBr into SBR composites also led to an increase in initial *σ* (when compared with the reference sample without BmPyBr). However, this effect was not as obvious as in the case of GnPs-filled composites. Probably, such result could be attributed to the different mode of interactions between a partially planar BmPyBr molecule with the flat GnPs particles or the spherical CB forms.

Additionally, the relaxation behavior of samples containing 3 or 5 phr of the filler was studied under shear deformation mode. The values of modulus *G* determined as a function of time were fitted to the generalized Maxwell model [60,61].

**Figure 14:**
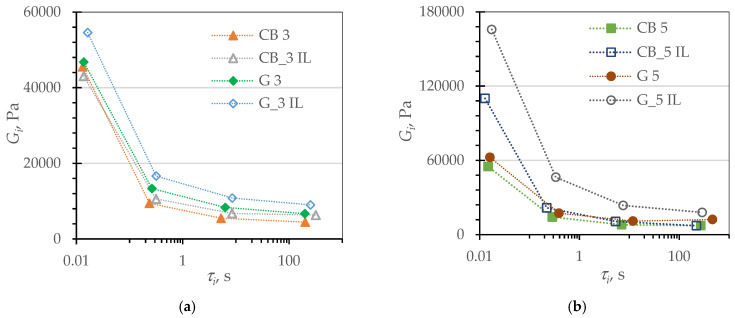
Discrete Maxwell relaxation spectra, Prony series coefficients: *G_i_* (relaxation moduli) and *τ_i_* (relaxation time) for SBR composites containing 3 (**a**) or 5 (**b**) phr of the filler.

The pairs of fitted *Gτ*_1_ parameters are showed in Figure 14. The values of calculated parameters of discrete relaxation spectra are compiled in Table S3. *G_i_* highlighted the differences in properties of composites containing GnPs and CB in the case of the short time of relaxation associated with the rotation of pending chemical groups present in the tested samples. The longer relaxation times (*τ*_1_, *τ*_2_) were achieved for the vulcanizates with GnPs compared with CB-filled composites, especially when the amount of filler yielded 5 phr. It can be attributed to π-π interactions formed between GnPs and phenyl groups of SBR. The incorporation of BmPyBr shortened *τ*_1_ and *τ*_2_ of the composites. Moreover, a strong decrease in *G_i_* were observed. The long-term relaxation times (*τ*_3_, *τ*_4_) of SBR vulcanizate containing 5 phr of GnPs and BmPyBr were also shortened. BmPyBr more strongly affected *τ_3_* and *τ*_4_ of CB-filled SBR vulcanizates. The long-term relaxation processes can be attributed to the movements of larger segements of polymeric chains or breaking of and subsequent rearrangement of physical cross-links present in the composite. Nanoparticles in composites can affect the transition of the relaxing rubber from one to another stage of relaxation. Both fillers, CB and GnPs, reduced the relaxation times describing filled sample, compared with neat SBR composite.

### 3.4. Viscoelastic Properties of SBR Composites. Payne’s Effect

**Scheme 2:**
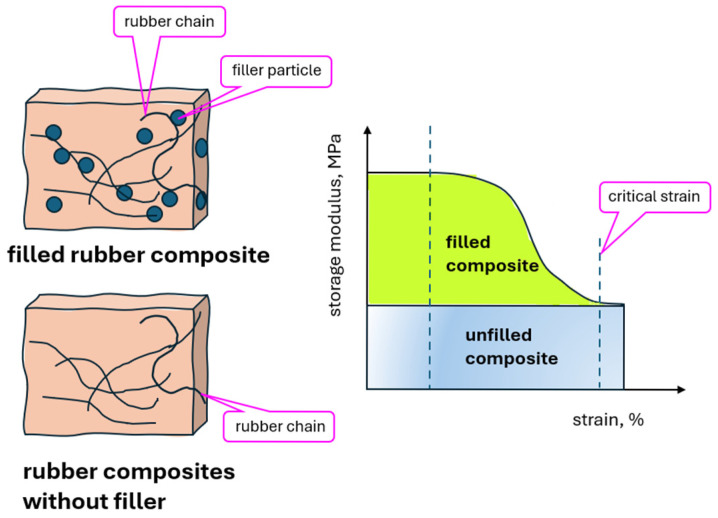
Viscoelastic properties of filled/unfilled rubber composites.

In this paper, two nano-sized carbonaceous fillers with different shapes and aspect ratios of particles were selected as additives, affecting the dynamic mechanical behavior of cured SBR rubber. The viscoelastic behavior of rubber composites can be influenced by interphase contact between the solid surface of filler and rubber chains. Nanostructured particles of filler with high surface area participate in the formation of a larger interphase with polymer macromolecules [71]. The Payne effect explains the differences in viscoelastic properties of filled/unfilled rubber composites (Scheme 2). CB belongs to active fillers, causing the strengthening of the mechanical properties of the filled and cured elastomer [45]. Similarly, nanoparticles of GnPs can influence the enhancement of rubber properties [75].

To estimate the effect of CB, GnPs and BmPyBr, the dynamic mechanical properties at room temperature such as complex modulus (G*), storage shear modulus (*G*′) and loss shear modulus (*G*′„) were determined and analyzed. The above-mentioned parameters calculated for the linear viscoelastic region (amplitude sweep tests) are compiled in Table 10 and Table 11. Here, 5 phr of CB or GnPs led to an increase in G* and *G*′ (almost four times) when compared with the unfilled sample.

The dynamic mechanical and viscoelastic response of filled rubber is complicated due to the fact that the enhancement of *G*′ is affected by various phenomena, i.e., mainly cross-link density of the composites and filler–filler and filler–rubber interactions (connected with filler’s presence in polymer, such as hydrodynamic effect of solid and rigid particles). The tendency to form a structure of filler particles in the elastomer matrix is one of the factors influencing the reinforcing effect. The solid particles can be linked together through physical interactions between functional groups present on the filler’s surface. CB’s ability to create its own structure is well known [76], but according to literature, such effect is observed for a higher concentration of filler [31]. In the case of low loading of active filler introduced into the rubber matrix, the uniform dispersion, strong polymer–filler interactions and the occlusion of the rubber chains on the filler’s surface are more strongly responsible for the effective reinforcement of polymer. Concisely, the occluded rubber is shielded from deformation and therefore increases the effective filler content [77]. The amount of polymer chains being adsorbed on solid particles’ surface and with restricted mobility is mostly affected by concentration, specific surface area and surface chemistry of the filler. *G*′ and G* analysis (Table 10 and Table 11) showed higher values of *G*′ and G* for composites with 5 phr of filler, compared with samples with lower filler loadings (1 or 3 phr). This revealed a higher amount of occluded rubber. Moreover, as the values of cross-link density (calculated from equilibrium swelling in toluene) showed no significant differences after the incorporation of a higher amount of both fillers, CB or GnPs, and BmPyBr (Figure S10), it can be concluded that the significantly higher values of the *G*′ for 5 phr of CB and GnPs resulted not from the differences in the stiffness of the cross-linked network but rather from the polymer–filler interactions. The enhancement of the elastic properties is more strongly observed for the nano-sized filler with higher aspect ratio and plate-like shaped particles. The reduction in the *G*′ and G* confirmed the role of filler–elastomer interactions in strengthening the samples containing, additionally, BmPyBr. The type and number of active centers on the filler’s surface influences the formation of filler–polymer interactions. It can be precisely adjusted through the modification of the filler’s surface. BmPyBr, used to prepare composites, being a low molecular compound, can easily diffuse to the surface of the fillers causing these active sites to be blocked. In this way, filler–polymer interactions are limited, leading to the decrease in the *G*′ and G* compared with filled SBR composites without BmPyBr. Due to the presence of the solid phase in the polymer composite influencing the dissipation of deformation energy, a strong increase in *G*′„ was observed after the introduction of a larger amount (5 phr) of both fillers: CB or GnPs.

**Figure 15:**
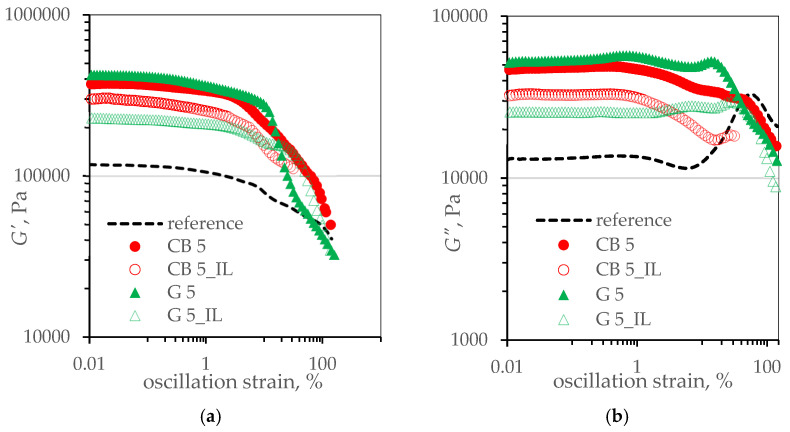
Storage shear modulus (*G*′) (**a**) and loss shear modulus (*G*′′) (**b**) of SBR vulcanizates as a function of oscillation strain measured at 25 °C; applied angular frequency 10 rads^−1^.

The viscoelastic behavior of the studied samples was analyzed more deeply. *G*′, *G*′„ and tanδ were determined for oscillation strain higher than linear viscoelastic region (Figures S16–S19). It is clear that the meaningful reinforcing effect visible only for 5 phr of CB loading led (for all CB loadings) to the reduction in *G*′. Moreover, the presence of BmPyBr in CB-filled composites reduced *G*′ and influenced the energy dissipation in vulcanizates, causing the *G*′„ changes. In the case of the SBR vulcanizates containing 3 or 5 phr of GnPs and BmPyBr, the opposite effects on the *G*′ were observed. It should be kept in mind that BmPyBr can improve the miscibility of GnPs in SBR, leading to re-aggregation of the filler and its better dispersion in matrix. This statement was proved by SEM (Figure 6). Furthermore, it resulted in the enhancement of the elastic properties of the composite (e.g., sample with BmPyBr and 3 phr of GnPs). Otherwise, BmPyBr covered the active sites on GnPs’ surface, making filler–polymer interactions impossible, thus leading to reduction in *G*′ (as it took place in composite containing BmPyBr and filled with 5 phr of GnPs). It should be emphasized that after the incorporation of 5 phr of CB or GnPs, similar enhancement of composites’ elastic modulus was observed (Figure 15). When SBR vulcanizate containing BmPyBr and 5 phr of CB is discussed, the occurrence of strong decrease in *G*′ at a lower oscillation strain (as the oscillation strain is increased) ought to be mentioned. A more significant decrease in *G*′ after the addition of BmPyBr was observed in the case of SBR composites filled with GnPs rather than with CB.

**Figure 16:**
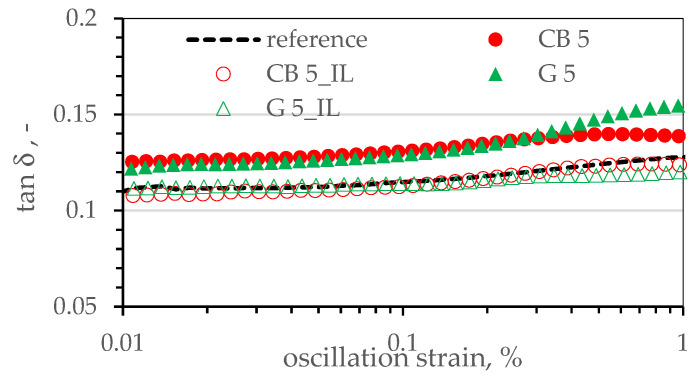
Loss factor (tan*δ*) of SBR vulcanizates as a function of oscillation strain measured at 25 °C; applied angular frequency 10 rads^−1^.

The presence of 5 phr of CB affected the damping properties of the SBR vulcanizates (Figure 16 and Figure S20). Better energy dissipation of the filled rubber composite was characterized by increasing tan*δ* values determined for the linear viscoelastic region (from 0.112 ± 0.001 for unfilled composite to 0.128 ± 0.001 in the case of the sample with 5 phr of CB). When the loading of CB was lower and in the presence of BmPyBr, no significant differences in composites’ tan*δ* were observed. Then, tan*δ* was in the range from 0.107 ± 0.001 (when specimen contained only 1 phr of CB) to 0.114 ± 0.002 (for composite with 3 phr of CB and IL). The improvement of damping properties was also noticed for the composite filled with 5 phr of GnPs. Then, tan*δ* was equal to 0.125 ± 0.002 (Figure 16). In most cases, tan*δ* decreased when BmPyBr was presented in samples.

The reinforcing effect of various fillers can also be estimated based on the analysis of Payne’s effect [65]. Generally, the break-up of filler–filler structure and the desorption of elastomer chains from filler’s surface at higher values of oscillation strain results in the decrease in composites’ complex modulus (G*) measured as a function of oscillation strain. While the disintegration of filler–filler interactions usually occurs up to the 5–10% of samples’ deformation [78], the extraction of polymer chains from the bounded rubber as well as the destruction of the filler–elastomer interactions protrude at higher oscillatory deformation [78]. Complex moduli determined at 5%, 20% and 50% of the deformation amplitude and the decrease in G* after exceeding a specific deformation level in relation to G*_LVR_ (ΔG*) are presented in Table 12 and Table 13. Additionally, both tables show the difference between the complex modulus value measured for the linear viscoelastic range at first and second deformation cycle (ΔG*_LVR_). G* and *G*′ for second cycle of deformation are presented in Tables S4 and S5 in SI. The obtained results confirmed the occurrence of strong filler–elastomer interactions in the case of composites containing GnPs (higher ΔG*_LVR_ and higher decrease in complex modulus at 5, 20 and 50%). Furthermore, the incorporation of BmPyBr, in most cases, caused the decrease in ΔG*_LVR_ when compared to samples without this additive. The effect was visible for both fillers, CB and GnPs. This is another proof that the presence of BmPyBr in polymer composites affected the formation of bounded rubber there.

### 3.5. Thermal Properties of SBR Composites

**Figure 17:**
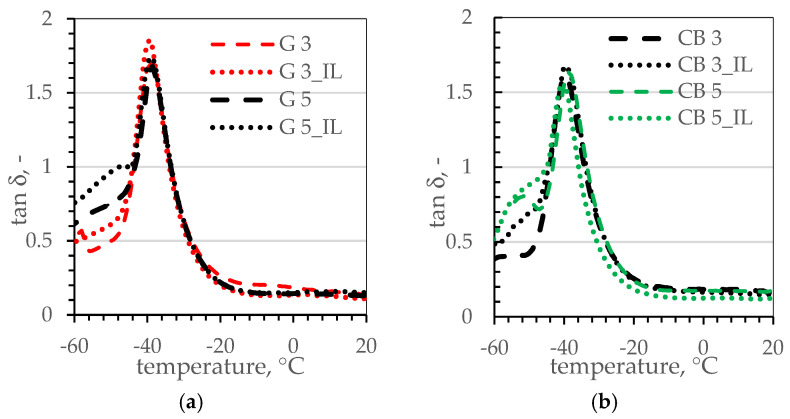
tan*δ* as a function of temperature for vulcanizates containing BmPyBr and GnPs (**a**) or CB (**b**).

To determine the glass transition temperatures (*T_g_*) of composites containing 3 or 5 phr of fillers, the oscillation temperature sweep tests were performed in a temperature range from −60 °C to 20 °C. The results are compiled in Table 14, tan*δ_Tg_* was read at the maximum of glass transition peak. The vulcanizates’ *T_g_*s were in a range of about −41 to −38 °C. The stronger shift in *T_g_* to lower temperatures was observed for the vulcanizate containing CB and BmPyBr. The higher amount of incorporated CB decreased the height of *T_g_* peak, leading to the lower values of tan*δ_Tg_*. It is attributed to the filler–elastomer interactions influencing the mobility of the chains in the glass transition region. Such effect was even stronger for CB than for GnPs (Figure 17). Moreover, GnPs-filled composites were characterized by wider glass transition peak when compared with samples with CB.

The incorporation of BmPyBr into composites filled with CB or GnPs influenced the values of tan*δ* above *T_g_* (Figure 17).

The values of storage and loss shear modulus (respectively, *G*′ and *G*′„) determined as a function of temperature for SBR vulcanizates containing both investigated fillers are presented in Figure S21.

## 4. Conclusions

The presence of ionic liquid (4-methyl-1-butylpyridinium bromide, namely BmPyBr) in the SBR composites affected their viscoelastic properties when graphene nanoplatelets (GnPs) or carbon black (CB) were used as composites’ fillers. GnPs, having higher than CB aspect ratio of particles, led to a stronger increase in viscoelastic parameters such as complex viscosity and storage modulus. These remarks were more pronounced when filler’s amount yielded 5 phr and the SBR compounds were plasticized before curing (at processing temperature 80 °C). The incorporation of solid fillers, CB or GnPs, affected the energy dissipation, causing the increase in loss modulus of plasticized and uncured SBR. Both discussed fillers reinforced tensile strength and storage modulus of SBR composites as filler loading was increased. Filler’s activity parameter and reinforcing indexes confirmed stronger reinforcing effect of GnPs. These additives strongly influenced the relaxation behavior of SBR composites as well.

Rubber composites with 5 phr of the filler modified in bulk with BmPyBr for rubber mixtures revealed better processability of SBR compounds. Additionally, GnPs were more homogenously dispersed within polymer in the presence of BmPyBr. Due to that, storage modulus of uncured SBR compositions increased. Flat carbon sheets of GnPs were more susceptible to interact with BmPyBr than CB particles. The interactions between partially flat BmPyBr particles and GnPs prevented, to some extent, blocking the interaction of the filler with the rubber chains. This, in turn, led to the reduction in Payne’s effect related to rubber–filler interactions. The presence of BmPyBr facilitated the relaxation of filled SBR composites in the force field. Although BmPyBr did not significantly affect curing of SBR or the amount of covalent cross-links formed in the polymer network, it slightly shortened pre-curing time.

## Figures and Tables

**Table 1:** Recipes for the compounds (the values are in phr (parts per hundred parts of rubber)).

	Name	Reference	CB	CB_IL	G	G_IL
Ingredients						
Rubber		100	100	100	100	100
Sulfur		2	2	2	2	2
MBTS		0.5	0.5	0.5	0.5	0.5
DPG		0.5	0.5	0.5	0.5	0.5
Carbon black		-	1–5	1–5	-	-
GnPs		-	-	-	1–5	1–5
Ionic liquid		-	-	0.23–1.15	-	0.23–1.15

**Table 2:** Surface energy of carbonous fillers before and after modification with the use of BmPyBr (*γ_s_*—surface energy, *γ_d_* and *γ_p_*—dispersive and polar components of surface energy).

Filler	*γ_s_* mN m^−1^	*γ_d_* mN m^−1^	*γ_p_* mN m^−1^
G	20.7	20.6	0.1
G_IL	30.2	24.6	5.6
CB	29.1	27.9	1.2
CB_IL	35.4	24.8	10.7

**Table 3:** The viscosity of uncured rubber compound after 2 min of plasticization at 80 °C (η*_max_ and *η*′*_max_*—maximum values of complex and dynamic viscosity, η*_2 *min*_ and *η*′_2 *min*_—mean values of complex and dynamic viscosity).

Sample’s Name	η*_max_, kPa·s	*η*′*_max_*, kPa·s	Δη*, kPa·s	Δ*η*′, kPa·s	η*_2 *min*_, kPa·s	*η*′_2 *min*_, kPa·s
reference	27.91	16.92	1.55	1.09	26.36 ± 0.64	15.83 ± 0.25
G 1	32.40	19.74	4.33	3.09	28.07 ± 0.59	16.65 ± 0.35
G 1_IL	31.47	20.21	1.93	3.43	29.54 ± 0.45	16.78 ± 0.08
G 3	34.16	20.91	3.5	3.17	30.66 ± 0.57	17.74 ± 0.29
G 3_IL	37.44	23.22	5.13	4.41	32.31 ± 0.52	18.81 ± 0.59
G 5	36.03	21.10	3.58	2.12	32.45 ± 0.62	18.98 ± 0.25
G 5_IL	37.45	22.19	3.67	3.28	33.78 ± 0.51	18.91 ± 0.08
CB 1	30.95	18.32	3.46	1.89	27.49 ± 0.69	16.43 ± 0.12
CB 1_IL	32.23	18.49	4.09	2.21	28.14 ± 0.67	16.28 ± 0.25
CB 3	34.01	21.83	4.42	4.43	29.59 ± 0.62	17.40 ± 0.42
CB 3_IL	34.23	19.04	4.48	2.43	29.75 ± 0.41	16.61 ± 0.06
CB 5	34.49	21.63	3.75	3.39	30.74 ± 0.73	18.24 ± 0.37
CB 5_IL	34.11	20.47	3.75	3.08	30.36 ± 0.69	17.39 ± 0.27

The parameters were calculated as follows: Δη* = η*_max_ − η*_2 *min*__._; Δ*η*′ = *η*′*_max_* − *η*′_2 *min*__._

**Table 4:** Characteristics of molten at 80 °C rubber compound at applied shear rate of 300 s^−1^ (*K*—consistency index; *n*—exponent of power law equation; η*—complex viscosity; r^2^—correlation coefficient).

Sample’s Name	*K*, kPas	*n*, -	r^2^	η* at 300 s^−1^, Pas
reference	53.61	−0.736	0.992	646.3
G 1	56.83	−0.739	0.998	656.0
G 1_IL	59.02	−0.748	0.991	654.9
G 3	58.49	−0.745	0.992	654.3
G 3_IL	59.39	−0.752	0.993	644.0
G 5	63.90	−0.753	0.993	687.1
G 5_IL	56.40	−0.763	0.997	576.2
CB 1	54.53	−0.738	0.990	641.9
CB 1_IL	61.09	−0.751	0.994	667.1
CB 3	57.85	−0.745	0.992	662.4
CB 3_IL	58.23	−0.748	0.993	626.7
CB 5	59.09	−0.745	0.991	665.0
CB 5_IL	50.00	−0.730	0.998	588.9

**Table 5:** Viscoelastic properties of SBR rubber compounds in the linear viscoelastic range (*G*′—storage shear modulus, *G*′„—loss shear modulus, tan*δ*—loss factor).

Sample’s Name	*G*′*_LVR_*, kPa	*G*′„ *_LVR_*, kPa	tan*δ* _LVR_, kPa
reference	66.13 ± 2.21	49.83 ± 0.93	0.75 ± 0.03
G 1	70.97 ± 1.97	52.32 ± 1.10	0.74 ± 0.02
G 1_IL	76.37 ± 1.56	52.70 ± 0.26	0.69 ± 0.01
G 3	78.56 ± 1.73	55.74 ± 0.90	0.71 ± 0.01
G 3_IL	82.49 ± 1.66	59.09 ± 1.85	0.72 ± 0.03
G 5	82.67 ± 2.21	59.63 ± 0.80	0.72 ± 0.02
G 5_IL	87.94 ± 1.78	59.39 ± 0.26	0.68 ± 0.01
CB 1	68.87 ± 2.28	52.10 ± 0.91	0.76 ± 0.03
CB 1_IL	72.08 ± 2.38	51.14 ± 0.79	0.71 ± 0.02
CB 3	75.16 ± 1.94	54.68 ± 1.33	0.73 ± 0.02
CB 3_IL	77.54 ± 1.58	52.17 ± 0.18	0.67 ± 0.01
CB 5	77.73 ± 2.45	57.29 ± 1.15	0.74 ± 0.02
CB 5_IL	78.19 ± 2.24	54.63 ± 0.85	0.70 ± 0.02

**Table 6:** Kinetic parameters characterizing curing process of SBR compounds containing 5 phr of CB or GnPs (*CR_max_*—cure rate at the maximum of the first derivative peak, *t*—time, *K*—rate constant at a particular temperature, *n*—the order of reaction, r^2^—correlation coefficient, *E_a_*—activation energy of rubber compound curing).

T. °C	CB 5						CB 5_IL					
	*CR_max_*	*t*, s	*K* × 10^−4^	*n*	r^2^	*E_a_* kJ Mole^−1^	*CR_max_*	*t*, s	*K* × 10^−4^	*n*	r^2^	*E_a_* kJ Mole^−1^
160	1.115	31.2	0.384	1.78	0.9901	75.78	0.72	26.4	0.798	1.58	0.9973	169.08
165	1.555	26.4	0.517	1.82	0.9913		1.07	22.8	1.676	1.52	0.9988	
170	2.048	26.9	0.679	1.87	0.9905		1.43	23.4	3.187	1.49	0.9998	
175	2.836	22.8	0.753	1.96	0.9923		1.80	20.4	4.568	1.49	0.9998	
180	3.613	19.8	1.016	2.03	0.9958		2.39	17.4	6.418	1.50	0.9995	
T. °C	G 5						G 5_IL					
	*CR_max_*	*t*. s	*K* × 10^−4^	*n*	r^2^	*E_a_*. kJ Mole^−1^	*CR_max_*	*t*. s	*K* × 10^−4^	*n*	r^2^	*E_a_*. kJ Mole^−1^
160	0.743	27.6	0.890	1.58	0.9970	128.61	0.479	30.5	0.188	1.75	0.9942	212.95
165	0.981	27.0	1.131	1.58	0.9977		0.635	28.0	0.236	1.81	0.9993	
170	1.404	24.0	1.907	1.59	0.9989		0.917	25.2	0.747	1.67	0.9962	
175	1.860	23.9	2.581	1.61	0.9995		1.181	21.6	1.134	1.66	0.9974	
180	2.539	18.6	4.239	1.61	0.9997		1.622	18.5	2.248	1.63	0.9940	

**Table 7:** Filler’s activity (*SE300/SE100*) and filler’s reinforcing index (*RI_TS_*) determined for CB or GnPs-filled SBR composites.

Sample’s Name	*SE300/SE100*, -	*RI_TS_*_,_ %	Sample’s Name	*SE300/SE100*, -	*RI_TS_*_,_ %
CB 1	2.23	1.3	G 1	1.97	1.2
CB 1_IL	2.05	1.3	G1_IL	2.45	1.3
CB 3	2.11	5.1	G 3	1.97	4.9
CB 3_IL	2.10	4.3	G 3_IL	1.90	4.1
CB 5	2.30	9.9	G 5	2.20	9.1
CB 5_IL	1.84	6.9	G 5_IL	2.22	10.8

**Table 8:** Size characteristics of filler aggregates in polymer composites containing 5 phr of GnPs or CB (x—diameter of aggregates, LA—the largest aggregates).

	SBR Composites	G 5	G 5_IL	CB 5	CB 5_IL
X, μm		%Number	%Number	%Number	%Number
<1		96.4	95.7	96.7	97.6
1 < x < 1.5		0.6	0.9	1.2	0.7
1.5 < x < 2		0.6	0.59	0.7	0.5
x > 2		2.5	2.9	1.4	1.1
LA		13.4	5.8	9.1	3.2

**Table 9:** Bound rubber content (*R_B_*) of SBR compounds filled with CB or GnPs.

Sample’s Name	*R_B_* %	Sample’s Name	*R_B_* %
CB 1	3.09	G 1	5.44
CB 1_IL	3.66	G1_IL	5.92
CB 3	3.98	G 3	2.90
CB 3_IL	2.85	G 3_IL	3.46
CB 5	4.15	G 5	3.65
CB 5_IL	1.06	G 5_IL	4.17

**Table 10:** Viscoelastic parameters of SBR composites filled with CB, measured for the linear viscoelastic region, LVR (*G*′—storage shear modulus, *G*′„—loss shear modulus, G*—complex modulus, RI_G__*_—reinforcing index).

Sample’s Name	*G*′*_LVR_*, kPa	*G*′„*_LVR_*, kPa	G* *_LVR_*, kPa	*RI*_G*_, -
reference	116.99 ± 0.53	13.14 ± 0.07	117.73 ± 0.52	n.a
CB 1	165.08 ± 1.18	17.67 ± 0.07	166.02 ± 1.17	0.014
CB 1_IL	148.91 ± 1.71	16.39 ± 0.16	149.81 ± 1.72	0.013
CB 3	149.70 ± 2.20	16.59 ± 0.16	150.61 ± 2.20	0.038
CB 3_IL	142.78 ± 1.74	16.32 ± 0.07	143.71 ± 1.73	0.037
CB 5	372.86 ± 1.80	47.54 ± 0.38	375.87 ± 1.78	0.160
CB 5_IL	297.57 ± 4.27	32.71 ± 0.19	299.36 ± 4.25	0.127

* values were calculated for the oscillation strain range 0.01–0.1% based on the amplitude sweep tests (temperature 25 °C, angular frequency 10 rad s^−1^).

**Table 11:** Viscoelastic parameters of SBR composites filled with GnPs, measured for the linear viscoelastic region, LVR (G′—storage shear modulus, G′„—loss shear modulus, G*—complex modulus, RI_G*_—reinforcing index).

Sample’s Name	*G*′*_LVR_*, kPa	*G*′„*_LVR_*, kPa	G* *_LVR_*, kPa	*RI*_G*_, -
reference	116.99 ± 0.53	13.14 ± 0.07	117.73 ± 0.52	n.a.
G 1	153.62 ± 1.95	18.86 ± 0.09	154.78 ± 1.94	0.013
G 1_IL	127.15 ± 1.09	12.81 ± 0.18	127.79 ± 1.07	0.011
G 3	161.99 ± 0.28	17.76 ± 0.14	162.96 ± 0.28	0.042
G 3_IL	195.90 ± 1.63	23.26 ± 0.08	197.28 ± 1.62	0.050
G 5	420.60 ± 2.74	52.66 ± 0.40	423.89 ± 2.67	0.180
G 5_IL	226.48 ± 1.53	25.57 ± 0.04	227.92 ± 1.53	0.097

* values were calculated for the oscillation strain range 0.01–0.1% based on the amplitude sweep tests (temperature 25 °C, angular frequency 10 rad s^−1^).

**Table 12:** Viscoelastic properties of SBR composites containing carbon black (ΔG*—decrease in the complex modulus at 5, 20, 50% of oscillation strain; ΔG*_LVR_—difference in G* measured for linear viscoelastic region between first and second cycle of samples deformation).

Sample’s Name	ΔG*_5%_, kPa	ΔG*_20%_, kPa	ΔG*_50%_, kPa	ΔG*_LVR_, kPa
reference	25.95	47.85	52.54	6.10
CB 1	27.24	53.18	96.42	42.91
CB 1_IL	25.02	55.77	64.62	49.38
CB 3	19.74	46.85	67.38	32.92
CB 3_IL	47.82	88.29	107.44	44.96
CB 5	95.8	205.07	262.26	283.15
CB 5_IL	93.28	175.1	205.49	195.23

The values were calculated as follows: ΔG*_5,20,50%_ = G*_LVR_ − G*_5,20,50%_; ΔG*_LVR_ = G*_LVR (1st cycle)_ − G*_LVR (2nd cycle)_.

**Table 13:** Viscoelastic properties of SBR composites containing GnPs (ΔG*—decrease in the complex modulus at 5, 20, 50% of oscillation strain; ΔG*_LVR_—difference in G* measured for linear viscoelastic region between first and second cycle of samples deformation).

Sample’s Name	ΔG*_5%_, kPa	ΔG*_20%_, kPa	ΔG*_50%_, kPa	ΔG*_LVR_, kPa
reference	25.95	47.85	52.54	6.10
G 1	53.17	84.19	114.46	50.41
G 1_IL	60.19	76.40	94.50	14.89
G 3	7.54	31.46	59.86	107.06
G 3_IL	37.69	68.49	108.33	97.91
G 5	104.80	281.12	357.81	239.01
G 5_IL	39.14	70.39	117.85	127.04

The values were calculated as follows: ΔG*_5,20,50%_ = G*_LVR_ − G*_5,20,50%_; ΔG*_LVR_ = G*_LVR (1st cycle)_ − G*_LVR (2nd cycle)_.

**Table 14:** Glass transition temperature (*T_g_*) and values of tan*δ* at *T_g_* of SBR composites (tan*δ*_Tg_).

Sample’s Name	*T_g_*, °C	tan*δ*_Tg_, -
reference	−39.8	1.77
CB 3	−39.3	1.61
CB 3_IL	−39.4	1.68
CB 5	−38.9	1.64
CB 5_IL	−41.2	1.53
G 3	−38.6	1.70
G 3_IL	−39.4	1.86
G 5	−38.6	1.68
G 5_IL	−38.3	1.72

## Data Availability

Data are available on request.

## Supplementary Materials

The following supporting information can be downloaded at: https://www.mdpi.com/article/10.3390/polym17243271/s1, Figure S1: FTIR-ATR spectra of pristine fillers, BmPyBr and mixtures of GnPs or CB with BmPyBr; Figure S2: Complex viscosity (*η**) as a function of plasticization time at 80 °C for rubber compounds containing CB and BmPyBr; Figure S3: Complex viscosity (*η**) as a function of plasticization time at 80 °C for rubber compounds containing GnPs and BmPyBr; Figure S4: Dynamic viscosity (*η*′) as a function of plasticization time at 80 °C for rubber compounds containing CB and BmPyBr; Figure S5: Dynamic viscosity (*η*′) as a function of plasticization time at 80 °C for rubber compounds containing GnPs and BmPyBr; Figure S6: Complex viscosity (*η**) as a function of shear rate at 80 °C for rubber compounds containing CB; Figure S7: Complex viscosity (*η**) as a function of shear rate at 80 °C for rubber compounds containing GnPs; Figure S8: Storage shear modulus (*G*′) as a function of frequency at 80 °C for rubber compounds containing CB or GnPs. Applied oscillation strain 7%; Figure S9: Loss shear modulus (*G*′„) as a function of frequency at 80 °C for rubber compounds containing CB (a) or GnPs (b). Applied oscillation strain 7%; Figure S10: Cross-link density of SBR composites with various fillers loadings gray bar—reference sample, blue bar—sample with CB, empty blue bar—sample with CB and BmPyBr, red bar—sample with GnPs, empty red bar—sample with GnPs and BmPyBr; Figure S11: Elastic torque (*M*) as a function of curing time at various temperatures 160–180 °C for SBR containing 5 phr of CB; Figure S12: Elastic torque (*M*) as a function of curing time at various temperatures 160–180 °C for SBR containing 5 phr of CB and BmPyBr; Figure S13: Elastic torque (*M*) as a function of curing time at various temperatures 160–180 °C for SBR containing 5 phr of GnPs; Figure S14: Elastic torque (*M*) as a function of curing time at various temperatures 160–180 °C for SBR containing 5 phr of GnPs and BmPyBr; Figure S15: Stress at 100% strain (*SE100*) of SBR composites gray bar—reference sample, blue bar—sample with CB, empty blue bar—sample with CB and BmPyBr, red bar—sample with GnPs, empty red bar—sample with GnPs and BmPyBr; Figure S16: Storage shear modulus (*G*′) as a function of oscillation strain for SBR rubber mixtures filled with CB or CB in the presence of BmPyBr; Figure S17: Loss shear modulus (*G*′„) as a function of oscillation strain for SBR rubber mixtures filled with CB or CB in the presence of BmPyBr; Figure S18: Storage shear modulus (*G*′) as a function of oscillation strain for SBR rubber mixtures filled with GnPs or GnPs in the presence of BmPyBr; Figure S19: Loss shear modulus (*G*′„) as a function of oscillation strain for SBR rubber mixtures filled with GnPs or GnPs in the presence of BmPyBr; Figure S20: Loss factor (tan*δ*) as a function of oscillation strain for SBR rubber mixtures filled with CB or GnPs; Figure S21: Storage shear modulus (*G*′) determined as a function of temperature for vulcanizates containing GnPs (a) or CB (b); Figure S22: Loss shear modulus (*G*′„) determined as a function of temperature for vulcanizates containing GnPs (a) or CB (b); Table S1: Curing performance parameters for rubber composites; Δ*M* the increase in the elastic torque during curing (Δ*M* = *M_H_* − *M_L_* where *M_L_*—minimum value of elastic torque, *M_H_*—maximum value of elastic torque), *CRI*—curing rate index; Table S2: Content of noncovalent cross-links in polymer network (Δ*ν*); Table S3: Discrete Maxwell relaxation spectra. Prony series coefficients: G_i_ (relaxation moduli) and τ_i_ (relaxation time) and equilibrium modulus G_e_, r^2^—correlation coefficient; Table S4: Values of complex modulus (*G**), storage shear modulus (*G*′), loss shear modulus (*G*′„) and loss factor (*tanδ*) determined for linear viscoelastic region LVR during II cycle of deformation for samples containing CB and BmPyBr; Table S5: Values of complex modulus (*G**), storage shear modulus (*G*′), loss shear modulus (*G*′„) and loss factor (tanδ) determined for linear viscoelastic region LVR during II cycle of deformation for samples containing GnPs and BmPyBr.
