# Supplementary material for: Elastic Composites Containing Carbonous Fillers Functionalized by Ionic Liquid: Viscoelastic Properties
Source: Polymers (Basel). 2025 Dec 9;17(24):3271. doi: 10.3390/polym17243271 (PMC12736943; doi:10.3390/polym17243271)
Supplement: Supplementary file 1 [file polymers-17-03271-s001.zip › polymers-3972382-supplementary.pdf]

polymers

Article

Elastic Composites Containing Carbonous Fillers Functionalized by Ionic Liquid: Viscoelastic Properties

Magdalena Gaca *, Magdalena Lipińska

[1] Department of Chemistry, Institute of Polymer and Dye Technology, Lodz University of Technology, 16 Stefanowskiego Street, 90-537 Lodz, Poland
* Correspondence: magdalena.gaca@p.lodz.pl; Tel.: +48-42-631-32-04

Table S1. Curing performance parameters for rubber composites; *ΔM* the increase in the elastic torque during curing ($\Delta M = M_H - M_L$ where M_L—minimum value of elastic torque, M_H—maximum value of elastic torque), *CRI*—curing rate index.

Sample's name	ΔM, dNm	CRI, min^{-1}	Sample's name	ΔM, dNm	CRI, min^{-1}
reference	6.57	7.21	reference	6.57	7.21
CB 1	6.76	6.70	G 1	6.98	6.38
CB 1_IL	6.93	5.62	G 1_IL	7.01	5.91
CB 3	6.81	6.40	G 3	6.58	5.62
CB 3_IL	7.28	5.43	G 3_IL	6.80	5.28
CB 5	7.11	6.49	G 5	7.53	5.50
CB 5_IL	7.33	6.27	G 5_IL	7.41	5.93

Table S2. Content of noncovalent cross-links in polymer network ($\Delta\nu$).

Sample's name	$\Delta\nu$, $\times 10^{-5}$ mole cm^{-3}	Sample's name	$\Delta\nu$, $\times 10^{-5}$ mole cm^{-3}
CB 1	0,08	G 1	0,29
CB 1_IL	0,08	G 1_IL	0,06
CB 3	0,10	G 3	0,06
CB 3_IL	0,14	G 3_IL	0,04
CB 5	0,20	G 5	0,03
CB 5_IL	0,05	G 5_IL	0,03

Table S3. Discrete Maxwell relaxation spectra. Prony series coefficients: G_i (relaxation moduli) and τ_i (relaxation time) and equilibrium modulus G_e, r^2—correlation coefficient.

composite	G_i., Pa	τ_I, s	G_e, Pa	r^2
reference	18436.7	0.01336	618.6	0.9982
	3056.9	0.552309		
	2719.62	32.5874		
	4744.44	735.196		
CB 3	45539.2	0.013094	35132.0	0.9991
	9423.29	0.235811		
	5469.34	5.25577		
	4448.48	200.532		
CB 3_IL	43060	0.013724	35872.4	0.9989
	10580.1	0.314727		
	6754.11	8.47037		
	6322.19	316.101		
CB 5	55019.5	0.014834	46017.8	0.9990
	14128.3	0.286807		
	7918.48	7.04612		
	7210.38	270.686		
CB 5_IL	110011	0.01281	69727.9	0.9992
	21570.2	0.221177		
	10603.6	5.33736		
	7145.75	222.389		
G 3	46793.5	0.0137799	42173.8	0.9992
	13316.2	0.263367		
	8333.2	6.26608		
	6688.48	196.926		
G 3_IL	54592.1	0.016325	56009.9	0.9992
	16628.8	0.315993		
	10813.1	8.50396		
	9020.16	250.625		
G 5	62433.8	0.016067	45587.5	0.9988
	17308.7	0.388215		
	10911.9	11.8284		
	12157.8	464.503		
G 5_IL	165730	0.017472	111393.0	0.9990
	46330.8	0.337072		
	23537.4	7.54439		
	17886.4	289.237		

Table S4. Values of complex modulus (*G**), storage shear modulus (*G′*), loss shear modulus (*G″*) and loss factor (*tan δ*) determined for linear viscoelastic region LVR during II cycle of deformation for samples containing CB and BmPyBr.

Sample's name	G^*_{LVR}, kPa II cycle	G'_{LVR}, kPa II cycle	G''_{LVR}, kPa II cycle	tan δ, - II cycle
reference	111.63±0.46	111.07±0.47	11.13±0.06	0.100±0.001
CB 1	123.11±1.36	122.17±1.41	15.12±0.31	0.124±0.004
CB_IL 1	100.43±0.62	99.95±0.62	9.80±0.15	0.098±0.002
CB 3	117.69±0.64	116.95±0.65	13.21±0.15	0.113±0.001
CB_IL 3	98.75±1.06	97.70±1.02	14.33±0.40	0.147±0.003
CB 5	92.72±0.90	91.91±0.92	12.19±0.13	0.133±0.003
CB_IL 5	104.13±0.55	103.73±0.55	9.06±0.19	0.087±0.002

Table S5. Values of complex modulus (*G**), storage shear modulus (*G'*), loss shear modulus (*G"*) and loss factor (*tan δ*) determined for linear viscoelastic region LVR during II cycle of deformation for samples containing GnPs and BmPyBr.

Sample's name	G^*_{LVR}, kPa II cycle	G'_{LVR}, kPa II cycle	G''_{LVR}, kPa II cycle	tan δ, - II cycle
reference	111.63±0.46	111.07±0.47	11.13±0.06	0.100±0.001
G 1	104.37±0.43	103.73±0.45	11.55±0.11	0.111±0.002
G_IL 1	112.90±1.08	112.33±1.09	11.28±0.10	0.100±0.002
G 3	70.54±0.80	70.67±0.81	8.19±0.10	0.117±0.002
G_IL 3	99.37±0.16	98.91±0.16	9.50±0.03	0.096±0.001
G 5	184.88±0.71	183.19±0.73	24.93±0.33	0.136±0.002
G_IL 5	100.88±0.84	100.12±0.82	12.35±0.26	0.123±0.002

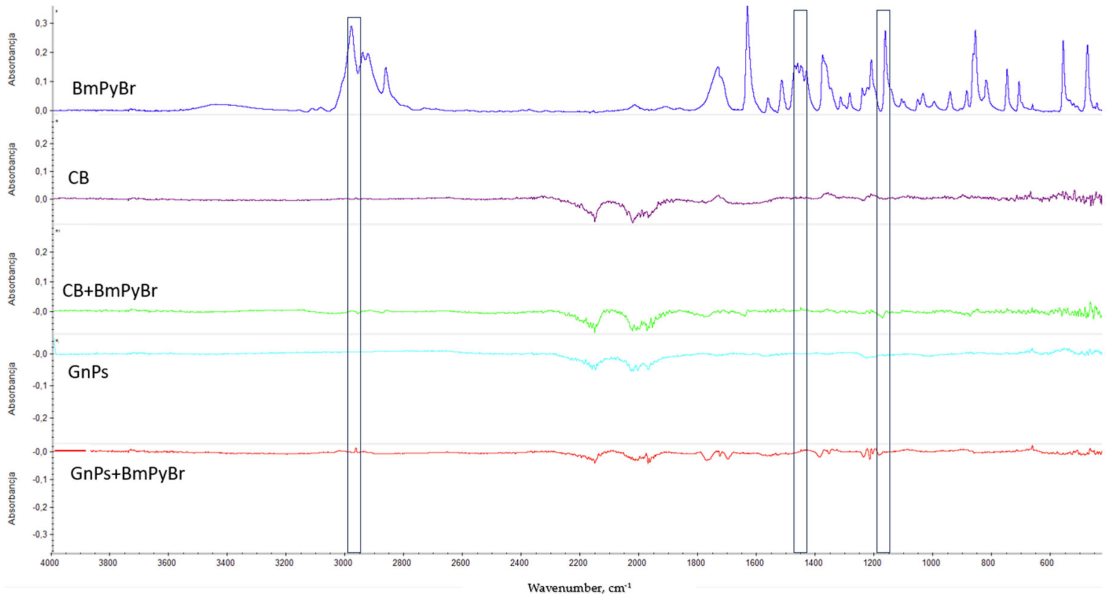

Figure S1. FTIR-ATR spectra of pristine fillers, BmPyBr and mixtures of GnPs or CB with BmPyBr.

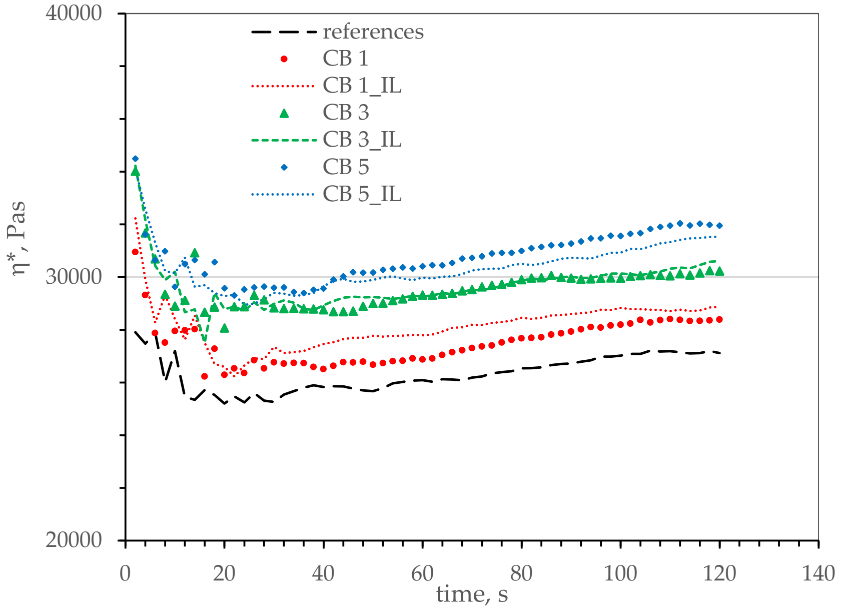

Figure S2. Complex viscosity (η*) as a function of plasticization time at 80°C for rubber compounds containing CB and BmPyBr.

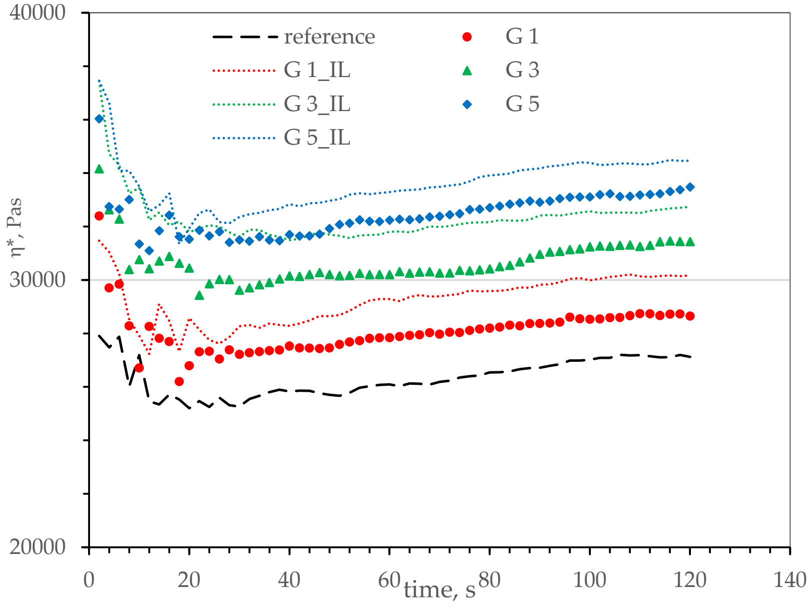

Figure S3. Complex viscosity (η*) as a function of plasticization time at 80°C for rubber compounds containing GnPs and BmPyBr.

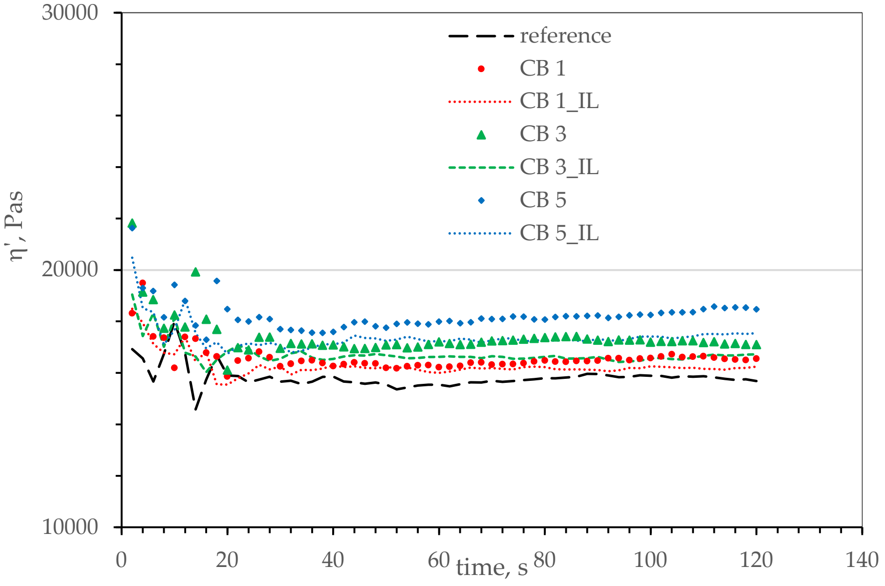

Figure S4. Dynamic viscosity (η') as a function of plasticization time at 80°C for rubber compounds containing CB and BmPyBr.

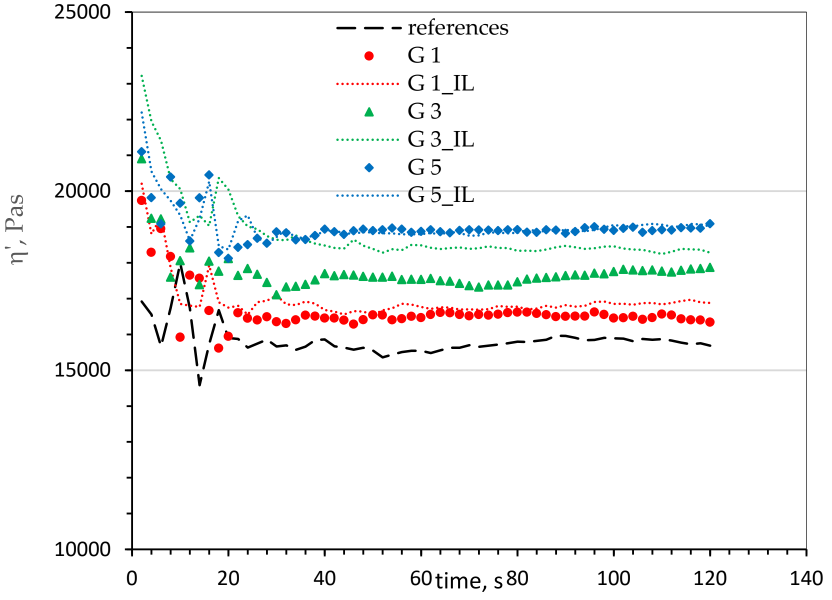

Figure S5. Dynamic viscosity (η') as a function of plasticization time at 80°C for rubber compounds containing GnPs and BmPyBr.

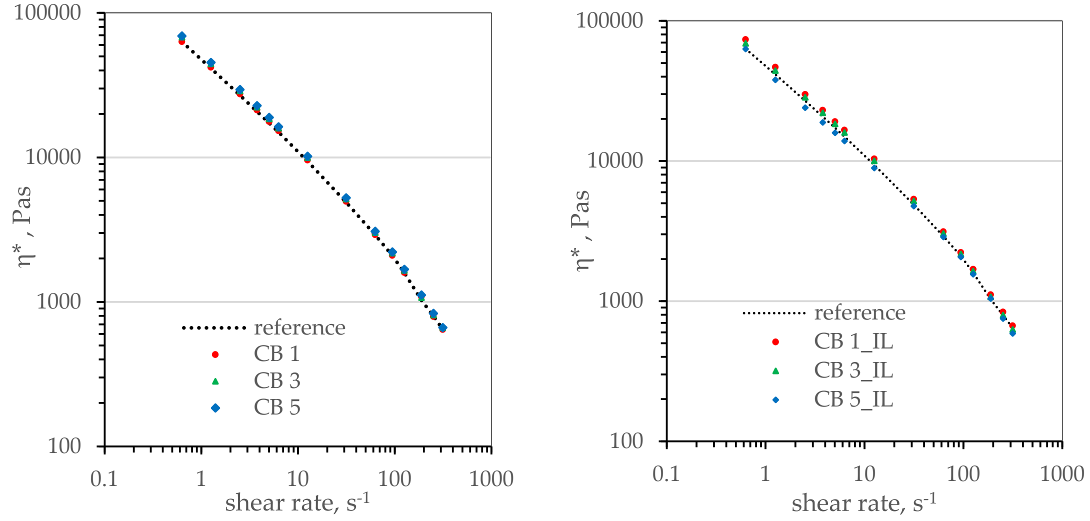

Figure S6. Complex viscosity (η*) as a function of shear rate at 80°C for rubber compounds containing CB.

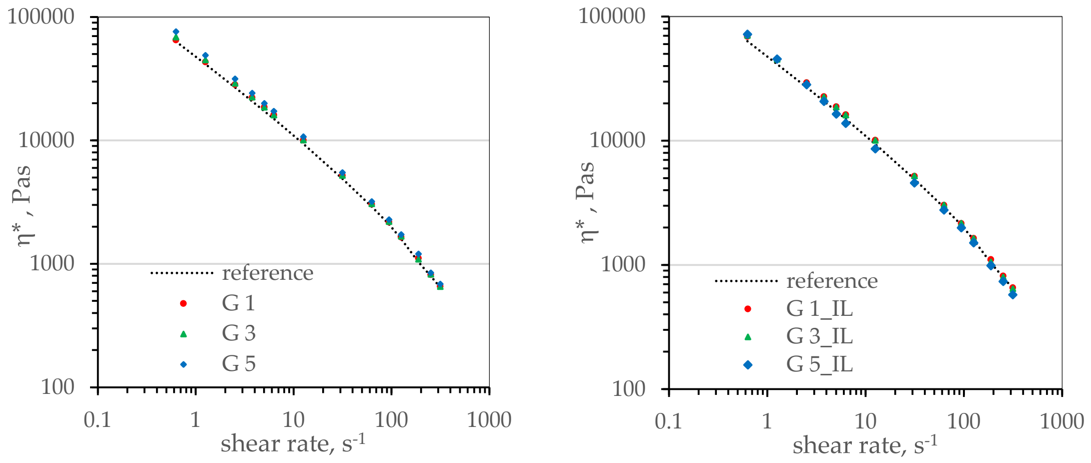

Figure S7. Complex viscosity (η*) as a function of shear rate at 80°C for rubber compounds containing GnPs.

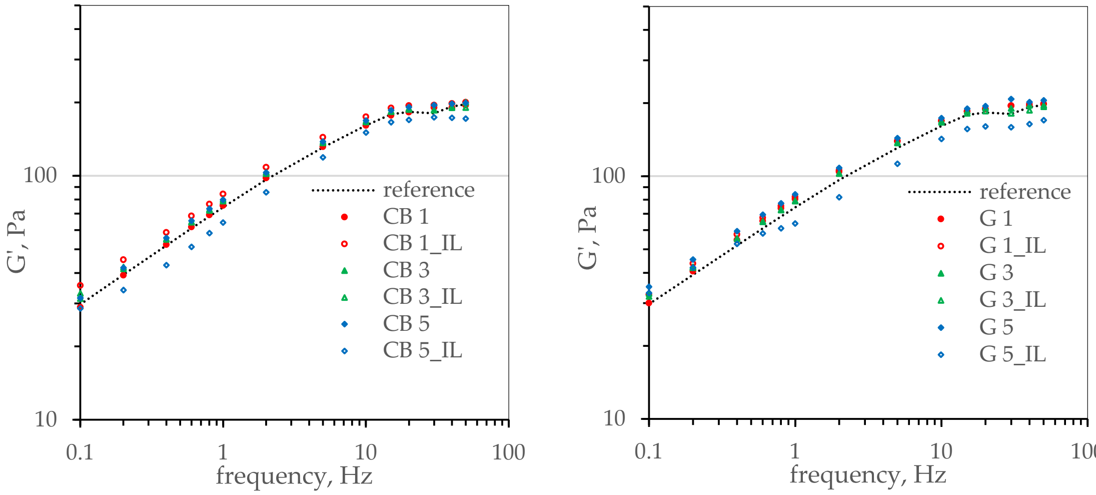

Figure S8. Storage shear modulus (*G′*) as a function of frequency at 80°C for rubber compounds containing CB or GnPs. Applied oscillation strain 7%.

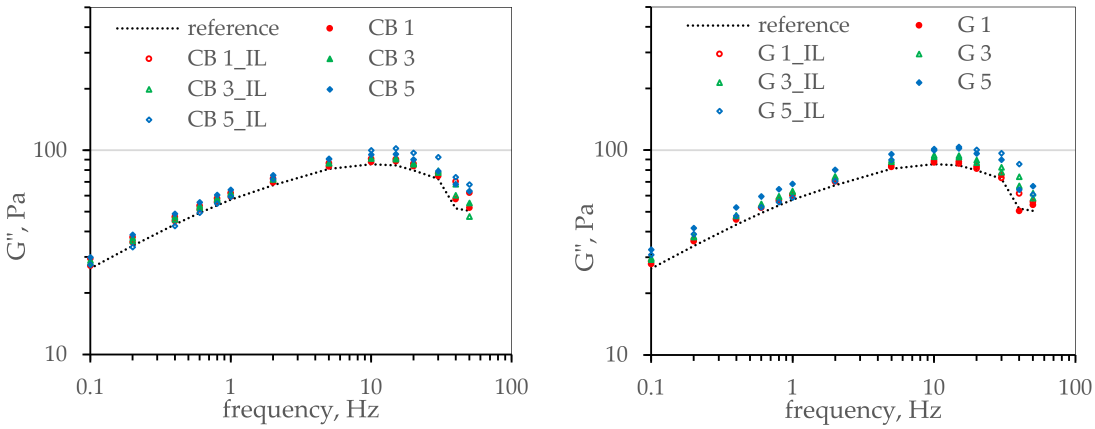

Figure S9. Loss shear modulus (*G′′*) as a function of frequency at 80°C for rubber compounds containing CB (a) or GnPs (b). Applied oscillation strain 7%.

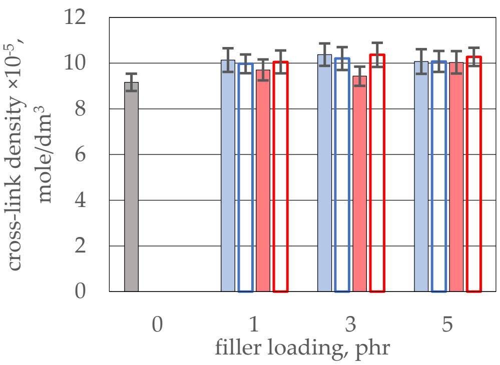

Figure S10. Cross-link density of SBR composites with various fillers loadings grey bar—reference sample, blue bar—sample with CB, empty blue bar—sample with CB and BmPyBr, red bar—sample with GnPs, empty red bar—sample with GnPs and BmPyBr.

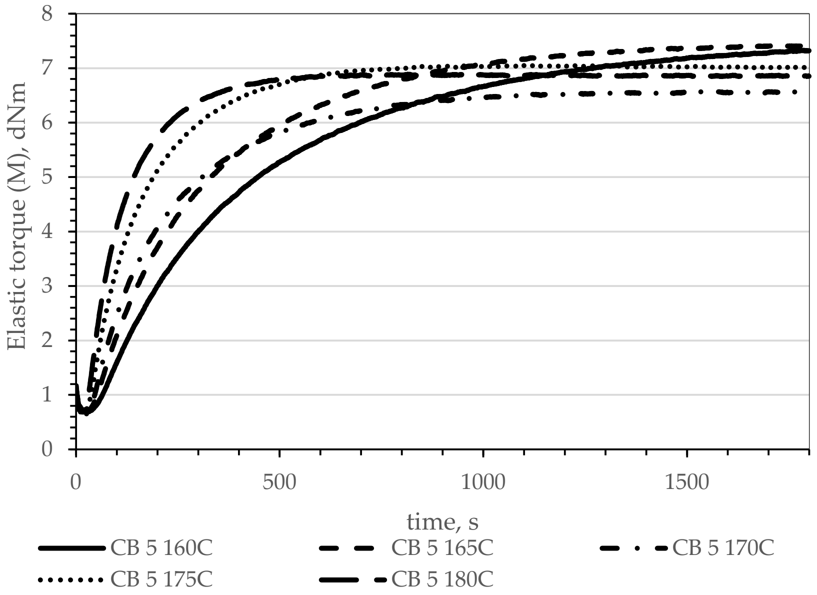

Figure S11. Elastic torque (*M*) as a function of curing time at various temperatures 160÷180°C for SBR containing 5 phr of CB.

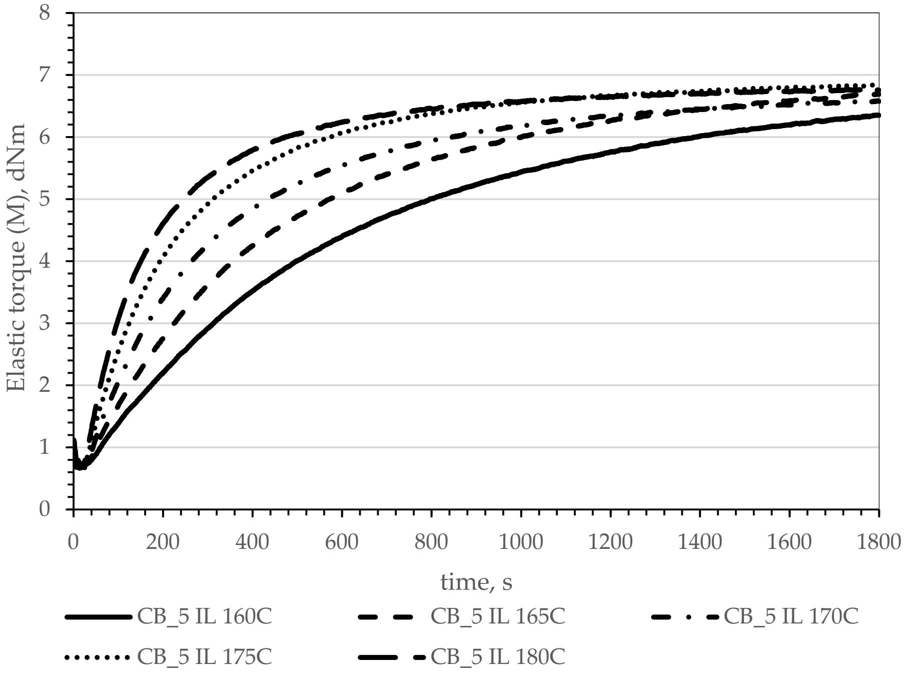

Figure S12.. Elastic torque (*M*) as a function of curing time at various temperatures 160÷180°C for SBR containing 5 phr of CB and BmPyBr.

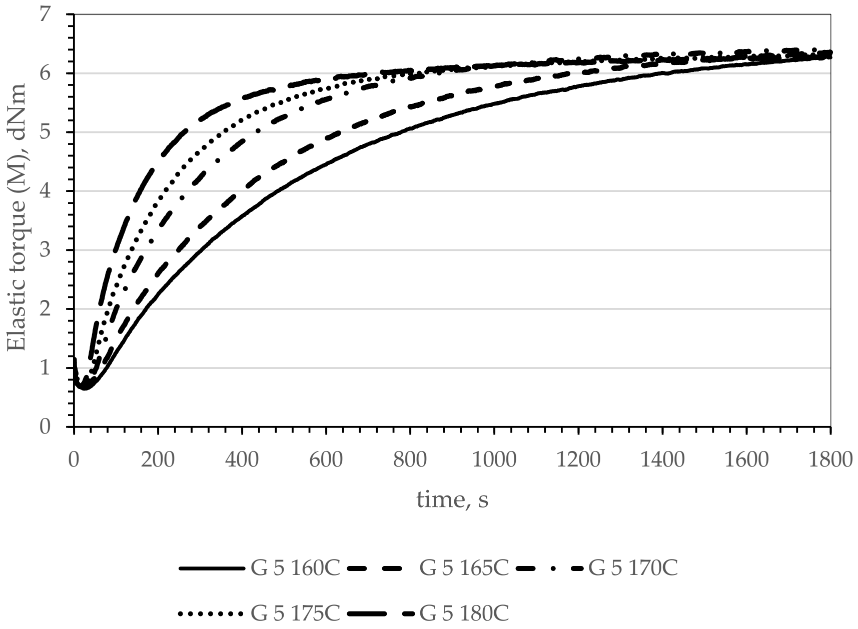

Figure S13. Elastic torque (*M*) as a function of curing time at various temperatures 160÷180°C for SBR containing 5 phr of GnPs.

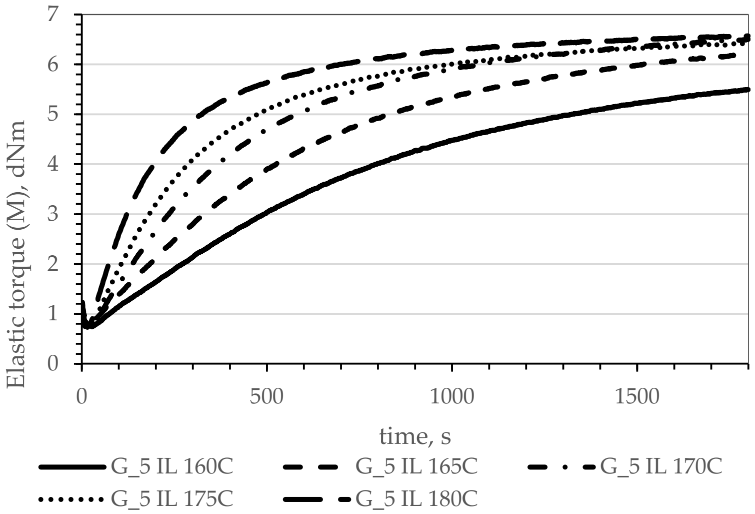

Figure S14. Elastic torque (*M*) as a function of curing time at various temperatures 160÷180°C for SBR containing 5 phr of GnPs and BmPyBr.

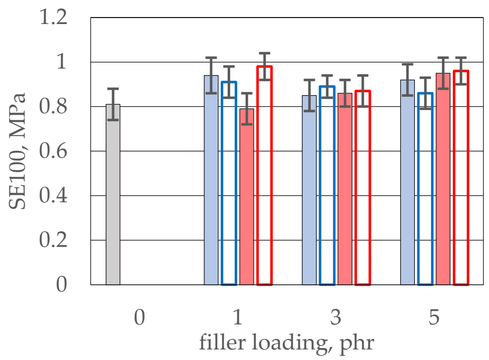

Figure S15. Stress at 100% strain (*SE100*) of SBR composites grey bar—reference sample, blue bar—sample with CB, empty blue bar—sample with CB and BmPyBr, red bar—sample with GnPs, empty red bar—sample with GnPs and BmPyBr.

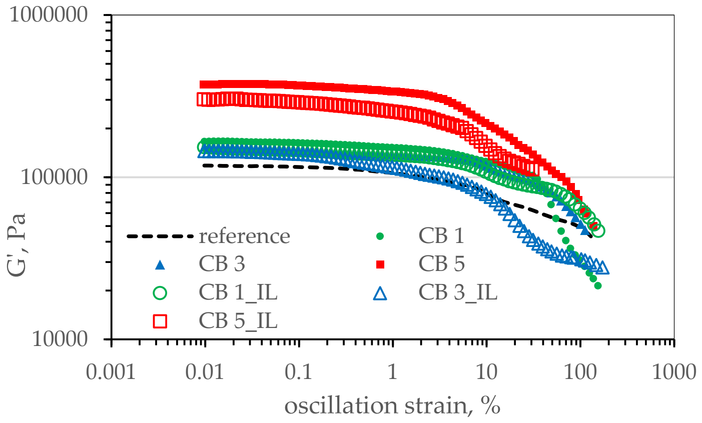

Figure S16. Storage shear modulus (*G'*) as a function of oscillation strain for SBR rubber mixtures filled with CB or CB in the presence of BmPyBr.

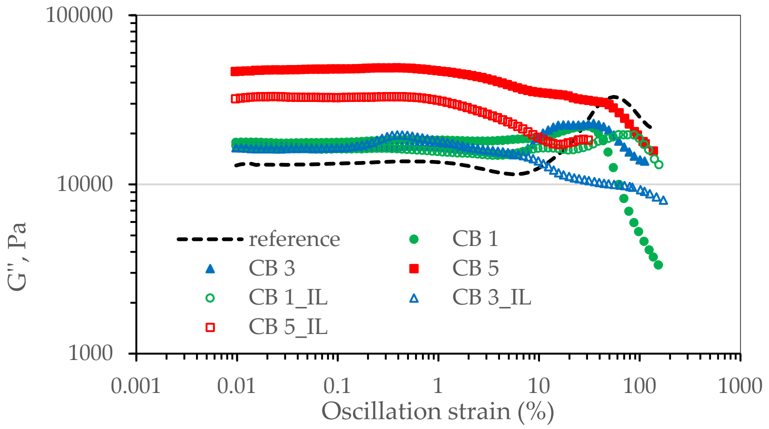

Figure S17. Loss shear modulus (*G''*) as a function of oscillation strain for SBR rubber mixtures filled with CB or CB in the presence of BmPyBr.

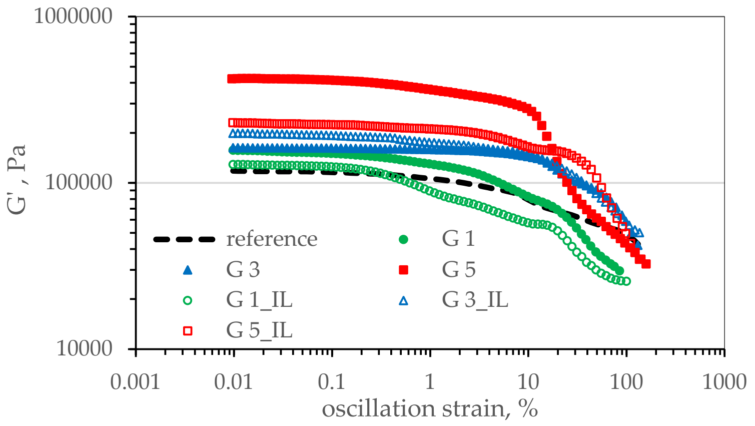

Figure S18. Storage shear modulus (*G′*) as a function of oscillation strain for SBR rubber mixtures filled with GnPs or GnPs in the presence of BmPyBr.

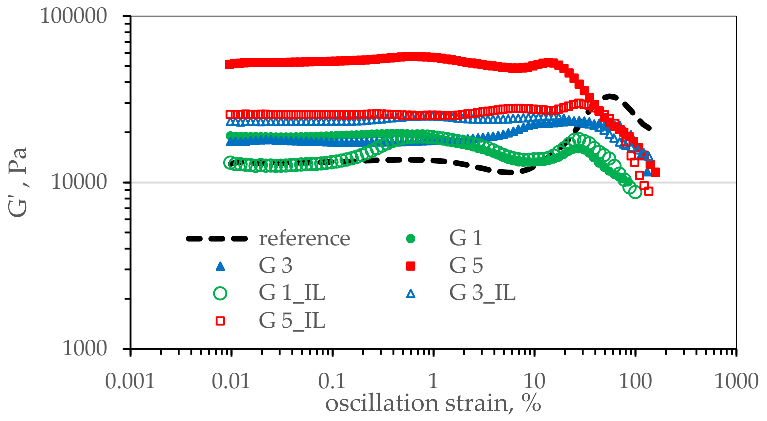

Figure S19. Loss shear modulus (*G″*) as a function of oscillation strain for SBR rubber mixtures filled with GnPs or GnPs in the presence of BmPyBr.

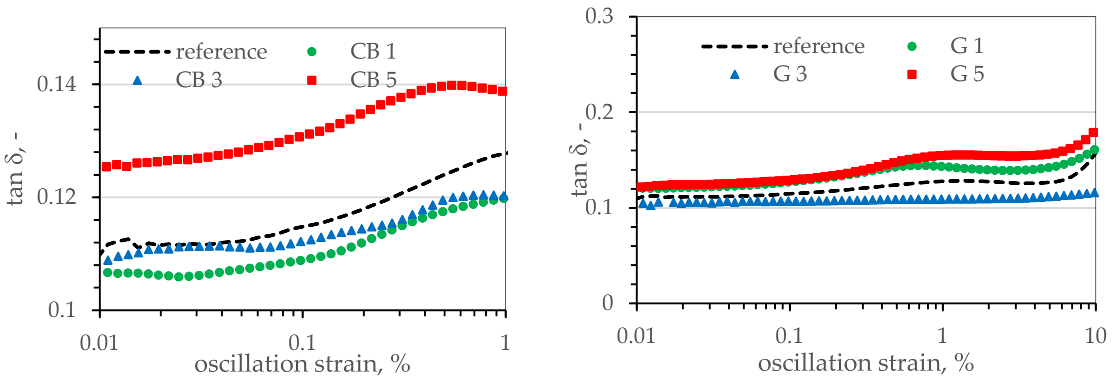

Figure S20. Loss factor (tan *δ*) as a function of oscillation strain for SBR rubber mixtures filled with CB or GnPs.

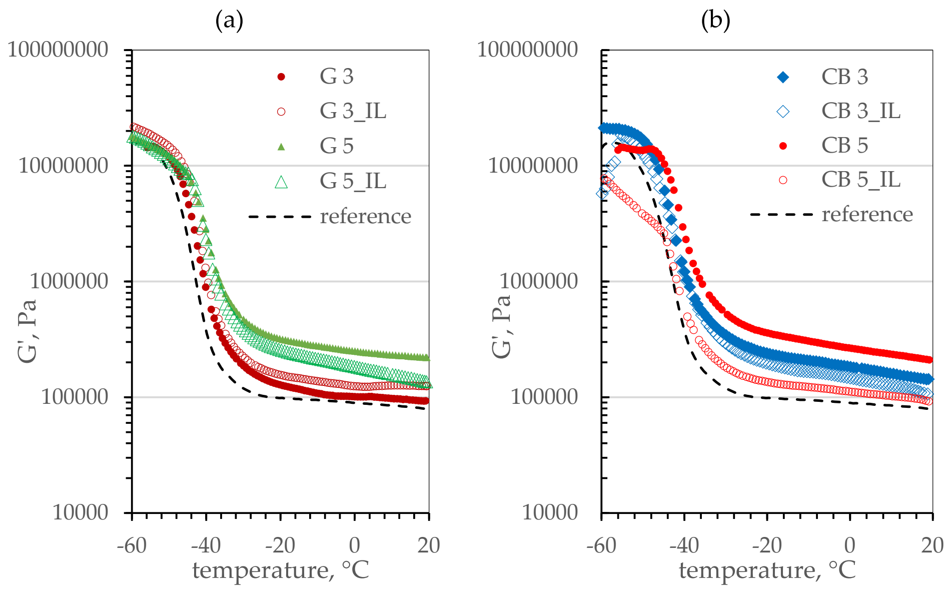

Figure S21. Storage shear modulus (*G′*) determined as a function of temperature for vulcanizates containing GnPs (a) or CB (b).

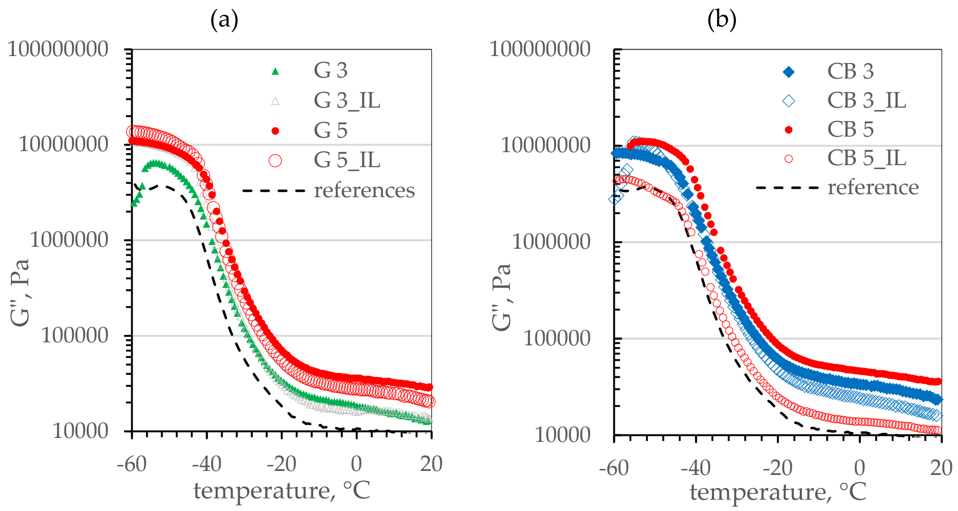

Figure S22. Loss shear modulus (*G″*) determined as a function of temperature for vulcanizates containing GnPs (a) or CB (b).
